# Impact of Threonine Supply in Early Ages on Gut Tissue Morphology, Liver Histology, and the Possible Changes in Leukocyte Numbers of Broilers

**DOI:** 10.3390/ani15030370

**Published:** 2025-01-27

**Authors:** Veronika Halas, Szilvia Áprily, József Nagy, Janka Turbók, Annamária Tischler, Nóra Katalin Szeli, Örs Petneházy, Orsolya Csötönyi, Judit Enyezdi, Virág Ács

**Affiliations:** 1 Institute of Physiology and Nutrition, Hungarian University of Agriculture and Life Sciences, Kaposvár Campus, H-7400 Kaposvár, Hungary; tischler.annamaria@uni-mate.hu (A.T.); szeli.nora@uni-mate.hu (N.K.S.); csotonyi.orsolya@uni-mate.hu (O.C.); 2 Institute of Animal Science, Hungarian University of Agriculture and Life Sciences, Kaposvár Campus, H-7400 Kaposvár, Hungary; 3 AVI-VET Ltd., H-7400 Kaposvár, Hungary; 4 Animal Health Diagnostic Directorate, Animal Health Diagnostic Department, National Food Chain Safety Office, H-7400 Kaposvár, Hungary; janka.turbok@gmail.com; 5 Medicopus Nonprofit Ltd., H-7400 Kaposvár, Hungary; 6 Pathodiagnostica Ltd., H-7623 Pécs, Hungary; enyezdij@pathodg.hu; 7 HUN-REN-MATE Mycotoxins in the Food Chain Research Group, Guba Sándor Street, H-7400 Kaposvár, Hungary; acs.virag@uni-mate.hu

**Keywords:** broilers, threonine, in ovo nutrition, hydrogel, compensation, performance, leukocytes, gut morphology

## Abstract

Delayed feed access post-hatch negatively impacts growth and intestinal development in broilers. Threonine (Thr) is known to support epithelial tissue development. This study evaluated the effects of early feeding strategies, including in ovo Thr administration and hydrogel provision, on broiler performance, gut and liver histomorphology, and leukocyte profiles. Control birds were fed immediately post-hatch, while others experienced a 48 h feed delay. One group received in ovo Thr on day 17 of incubation, while others were given Hydrogel^®^ with or without Thr during the first 48 h post-hatch. Immediate feed birds showed the best performance (*p* < 0.05), while early Thr supplementation (in ovo or hydrogel) partially mitigated the negative effects of delayed feed access. On day 21, birds treated with in ovo Thr had 4% higher body weight (BW) than untreated birds (*p* < 0.05). Birds given hydrogel post-hatch showed no significant BW differences compared to other groups (*p* > 0.05), but 48 h delayed, non-supplemented birds had 34.3% lower BW than immediate-fed birds (*p* < 0.05). Compensation was linked to improved gut architecture, though no significant effects were observed on leukocyte profiles or liver histology. In ovo Thr feeding is recommended to mitigate the adverse effects of a two-day delay in feed access for broiler chickens.

## Simple Summary

Broiler chickens travel long distances from the hatchery to the farms. This lag time may affect not just later performance, but also the development of the immune system. Threonine (Thr) is an essential amino acid that has key role in immune defense. Thus, the study aimed to examine the effects of some early nutritional practices such as in ovo feeding or provision of hydrogel on the morphology of the gut, histology of the liver, and the possible changes in white blood cell counts, with or without adding Thr in an early stage of life. Our results confirmed that birds without a feed deprivation period had the best performance and gut morphology, while threonine supplementation could partly compensate for the loss attributed to the post-hatch delay in feed access. There were no effects of early threonine feeding on the rate of white blood cells and histological parameters of the liver. The use of Hydrogel^®^ with or without Thr fortification during the post-hatch period improved the body weight at slaughter; thus, it can be recommended for use with broilers with delayed feed access.

## 1. Introduction

Fast-growing broilers must have a continuous and well-managed nutrient supply to realize the genetically determined slaughter weight at the target age. Recent findings have revealed that during the ‘critical periods’ of early development, environmental factors such as feed deprivation or nutrient supply significantly impact epigenetic perinatal programming, which can influence the entire life cycle [1]. The week around hatching is particularly sensitive; disturbances during this period may alter the signaling mechanisms of genes responsible for physiological processes; however, nutrient-induced alterations during this critical phase may also enhance compensatory mechanisms [2].

The hatching egg contains essential nutrients for embryo development, but the length of the hatching window as well as the time to the first feed post-hatch determines slaughter weight. It has been confirmed that in intensive broilers, even 24 h delay in feed access post-hatch reduces the body weight by 2–3%, while a longer time of feed deprivation (48–72 h) results in 5–8% less slaughter weight at 42 days of age [3]. During the early period of life, the embryo relies on the antibodies and nutrients of the albumen; however, dynamic changes in the egg yolk from continuous metabolism can influence the development of the chick [3]. In addition, other stressors, such as limited nutrients in the final period of incubation [4], as well as a wide hatching window (12–24 h), and delayed first feeding [5] can also harm the immune functions and vitality of broilers. Thus, mortality in the first week is 1.5–2.2 times higher if the access to the first feed is delayed by 48–72 h compared to birds provided solid feed within 24–36 h post-hatch [3].

To avoid these outcomes, different early feeding methods have been developed, such as offering hydrogel enriched with various nutrients like electrolytes (Na-, Ca-, K-chloride, Mg-sulphate), organic selenium, specific amino acids (leucine, isoleucine), as well as vitamin C, prebiotics, and probiotics [6], ensuring immediate feed access post-hatch (Patio system [7]), or providing nutrients in ovo [6]. Vitamins, probiotics, and amino acids can be supplemented as a complementary feed in a gel form by spraying the chicks in the incubator [6] so that gel consumption is carried out by pecking each other. Another option for early nutrition of hydrated gels is applying them in the chick box during transport. The benefits of jelly masses are avoiding dehydration by prompt water consumption [8] and providing probiotics to boost intestinal microbiota and compensate for environmental stressors. It has been well documented that immediate feed access resulted in higher final weight, better breast meat-to-carcass ratio [9], rapid intestinal development [10], greater duodenal villi surface, favorable gut microbiota, and prevention of pathogenic infections by boosting the immune system [11,12]. However, in practice, immediate post-hatch feeding is difficult to carry out. In ovo intervention was first applied in vaccination research in the late 1980s [13], and the technique was later used to provide nutrients, particularly carbohydrates [8]. Over the years, several experiments were carried out to determine the optimal timing and site of injection by introducing exogenous substances to the air sac, yolk sac, and amniotic fluid [14,15,16]. It turned out that amniotic fluid proteins, minerals, and hormones can be supplied to the late-term embryo; therefore, in ovo nutrient provision is a reliable non-invasive method to boost immature digestive systems around the first days of life [15]. Improving chick immunity is also possible by adding vitamins, prebiotics, or amino acids before hatching [17,18]. From the immunomodulatory point of view, amino acids such as methionine (Met), arginine (Arg), and threonine (Thr) contribute to lymphocyte proliferation and enroll monocytes and heterophils from the bone marrow [19,20,21].

According to Sirsat et al. [22], modern broiler strains require reevaluation of their nutrient provisions, and specific early-phase nutrition must be applied, particularly in the pre-hatch and the transitional post-hatch period. A specific feeding strategy that improves the digestive efficiency and/or resilience of birds improves the economics of poultry production. The individual amino acids have a prominent role in this respect. Threonine supplementation improved the morphological characteristics of the intestine, thereby providing a better and larger absorption surface, as confirmed by Gehad et al. [23]. The larger absorption surface supports the supply of nutrients, which on one hand can result in better growth performance and on the other hand, can improve feed efficiency. If the proportion of valuable meat parts is improved, a higher live weight can accompany a better slaughter yield. Gehad et al. [23] confirmed the benefits of in ovo feeding of threonine. However, it is likely that, in their study, chickens received feed immediately post-hatch, rather than after a delay of some days. In practice, immediate access to feed post-hatch is uncommon. Moreover, the literature is scarce on whether in ovo feeding can compensate for the detrimental effects of delayed feed access post-hatch. Pesti-Asboth et al. [24] have observed that hepatic tissue damage in intensive broilers, including structural alterations and inflammatory cell infiltration, becomes more pronounced with age. The liver is a key organ for nutrient metabolism, so it is crucial to evaluate whether dietary interventions (feed deprivation, in ovo Thr supply, or hydrogel supplementation with or without Thr) during the sensitive developmental stage affect liver physiological development. It is well known that pathological conditions such as heterophil granulocyte infiltration, vacuolization, mononuclear infiltration, or lipid accumulation in the liver compromise liver function, hindering the birds’ ability to achieve their genetic potential.

Hence, the present study aimed to investigate the effects of different early nutrition strategies, focusing on early Thr supply via in ovo feeding or hydrogel provision, compared to immediate post-hatch feeding. The study assessed their impact on the performance, gut histomorphology, liver histology, and differentiated leukocyte number of broiler chickens.

## 2. Materials and Methods

### 2.1. Hatching Protocol and Treatment Groups

The experiment was carried out at the Hungarian University of Agricultural and Life Sciences (MATE) Kaposvár Campus, Department of Farm Animal Nutrition by the Declaration of the Hungarian National Scientific Ethical Committee of Animal Experimentation for studies involving animals, protocol license number is SO/31/00444-2/2021.

A total of 1120 Ross 308 broiler eggs were involved in the study. Prior to hatch, the eggs were held in transport boxes at 17–18 °C for six days without rotation or extra humidification due to the short storage time. A PLM B1350 two-staged incubator was used for the hatching process with nine tray levels. Each level was equipped with a built-in measurement system for ventilation, humidity, and temperature between the levels. The hatching protocol was carried out according to the recommendations of the Aviagen Hatching Management Guide [25]; the dry bulb temperature and humidity were set at 37.9 ± 0.1 °C and 65 ± 3%, respectively (Table 1). The eggs were candled on days 10 and 17 to exclude infertile eggs or dead embryos. The fertility of eggs was calculated after candling by the following formula:

Fertility rate % = number of fertile eggs/total number of eggs set.

To compare the performance of birds receiving early feeding with those fed under ideal and practical conditions, the eggs were allocated to seven experimental groups (Table 2). Birds fed immediately post-hatch represented the ideal condition (Int_0 and IoS_0), while those fed after a 48 h delay represented the practical condition. In delayed feed groups, there were five treatments, as follows: Int_48 eggs had no in ovo intervention and the birds hatched in that treatment had no access to feed or water for 48 h; IoS_48 and IoT_48 eggs were treated with in ovo saline without or with Thr, respectively, and had no access to feed or water for 48 h; Int_48G and Int_48GT eggs had no intervention during hatching and had no solid feed in the first 48 h post-hatch, but immediate access to Hydrogel^®^ (Bábolna Feed Ltd., Nagyigmánd, Hungary) without Thr or with 5 g/kg Thr enrichment, respectively, in the transport boxes. The basal Hydrogel^®^ was composed of corn starch (30%), probiotic lactic acid bacteria (*Pediococcus acidilactici* (E1712) 1 × 10^10^ CFU/g), and vitamin C (5000 mg/kg), and contained 5.4 MJ AMEn/kg. According to the experimental design, two groups of eggs were injected with 0.5 mL of physiological saline (0.9 g/mL concentration of NaCl), either from the immediate (IoS_0) or from the delayed feed groups (IoS_48). This intervention was needed to evaluate possible stress caused by the needle puncture and saline administration. One group of eggs was treated with in ovo Thr, while another group received Thr during the first 48 h post-hatch. This was done to evaluate whether Thr supplementation at the late embryonic stage or shortly after hatching could mitigate the negative effects of feed deprivation in the first days of life.

### 2.2. In Ovo Intervention

The in ovo injection was carried out following the protocol of Uni and Ferket [26], with a 2 mL syringe and a 21-gauge needle. The injection procedure was performed in a sterile cabinet (ScanLaf, LaboGen Inc., Lillerød, Denmark) to prevent any microbiological contamination. Before the intervention, all eggs were cleaned with cotton wool dipped in an iodine solution. The eggs were then carefully drilled on the blunt side through the air chamber without reaching the shell membrane. Prior to the intervention, the position of the embryo was carefully checked. A 0.5 mL solution was then injected into each egg. The solution was either pure physiological saline (0.9% *w*/*v* NaCl) or a threonine solution containing 5 mg of Thr dissolved in 1000 mg of physiological saline. The injection was administered into the amniotic fluid without causing injury to the embryo. To avoid entry of pathogens, a sterile, plastic tape was applied, and the eggs were placed back into the incubator until day 21 of hatching.

### 2.3. Identification, Feeding Management, and Housing

The birds were weighted with gram precision on hatching day right after harvesting, and wings were tagged for individual identification. Birds in treatment Int_0 and IoS_0 were immediately placed in the barn, where a mash starter feed was offered ad libitum. The rest of the birds were stored in a transporter paper box (25 birds/box) and kept at 32 °C for 48 h in a separate room. Birds assigned to Int_48, InS_48, and IoT_48 received no feed supplementation, while in groups Int_G48 and Int_GT48, the birds had access to hydrogel with or without Thr fortification. On day two, all birds were weighed with gram precision and placed in the barn where the starter feed was freely available.

The broilers were randomly placed into floor pens (16 birds/pen, eight pens/treatment). Each pen represented an individual treatment group and was equipped with one feeder and one drinker. The housing management was set in compliance with Aviagen’s [27] recommendations for temperature, humidity, hours, and intensity of light.

A three-phase feeding program was applied as follows: between days 1 and 10, starter feed (mashed feed); between days 11 and 21, pelleted grower feed; and between days 22 and 35, pelleted finisher feed produced by the Department of Farm Animal Nutrition was offered to all birds. Each feed was formulated on a corn–soybean meal base. Nutritional content (dry matter, crude protein, fat, ash, calcium, and phosphorus) was determined by the University Lab Center of MATE according to the recommendations of the Association of Official Analytical Chemists (AOAC [28]).

The birds were fed ad libitum from self-feeders during the trial. One feeder was presented per pen. Drinking water was also available ad libitum. The analyzed composition of the feed is presented in Table 3.

### 2.4. Experimental Procedure

The live weight of the birds was measured at hatch (d1), and on days 3, 10, 21, and 35. The average daily gain (ADG) was calculated individually, while feed intake (FI) was recorded per pen for the time intervals by measuring the offered and remaining feed for each phase (on days 3, 10, 21, and 35). The feed conversion ratio (FCR) was also calculated per pen.

At hatch, 48 h later, and at d21 of the experiment, 10 birds from each treatment were used to collect blood, liver, and intestinal samples. For that purpose, the birds were slaughtered following carbon dioxide stunning in compliance with the relevant legal regulations (Council Regulation 1099/2009/EC). In the case of day-old and 3-day-old chicks, capillary blood samples were collected from the dorsal metatarsal vein and direct blood smears were prepared immediately from 10 birds/treatment. Similarly to day-old chickens, the 21-day-old birds were sampled for bloodwork, but this time blood samples were collected from the jugular vein into 5 mL tubes containing EDTA for further evaluation. Blood smears were prepared immediately after the collection process.

**Figure 1:**
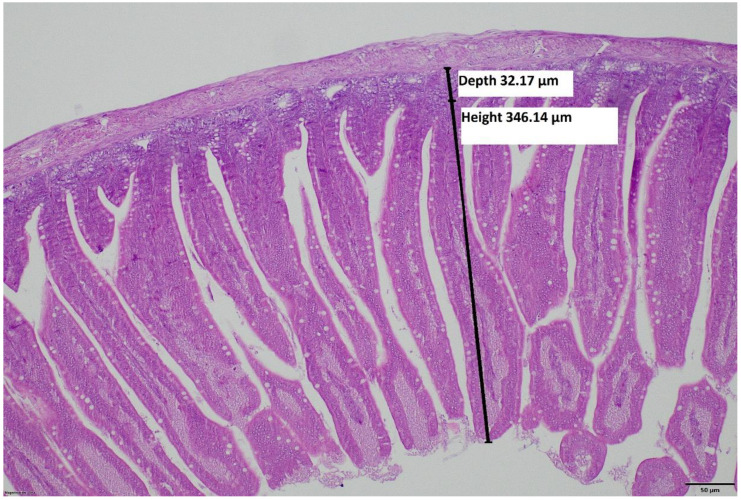
Transversal section of duodenum in a three-day-old chicken, H-E, 100×. Black lines represent the morphometric measurement of villus height and crypt depth.

To determine gut morphology through villus height (VH), crypt depth (CD), and villus/crypt ratio (VH/CD), tissue samples were collected from the duodenum, ileum, and colon (10 birds/treatment). Liver samples were also collected, and all samples were placed into a 10% neutral buffered formalin solution and processed in an Epredia™ Citadel 2000 Tissue Processor (Thermo Fischer Scientific, Waltham, MA, USA) to make paraffin-embedded blocks. Two-micrometer-thick paraffin sections were obtained in a rotary microtome unit (HistoCore BIOCUT, Leica Biosystems, Nussloch, Germany). These sections were de-waxed, rehydrated, and transferred to glass slides, and hematoxylin-eosin staining was performed according to standard histological techniques [29]. Histological sections of the liver samples were evaluated under light microscope (OLYMPUS BX43, OLYMPUS Corporation, Tokyo, Japan) at magnification 40–400×. Pathological injuries on the liver, such as leukocyte infiltration or hepatocellular lipid accumulation, were classified as none, mild, moderate, or marked lesions according to their severity with scoring from 0 to 3 according to [30,31,32,33,34]. For detailed description of these alterations please see File S1. Villus sizes and crypt depths were measured under light microscope (OLYMPUS BX43, OLYMPUS Corporation, Tokyo, Japan) at 100× magnification [35]. The measurement method is demonstrated in Figure 1. Each sample from each broiler was cross-sectioned three times, and three measurements were carried out on each section.

### 2.5. Statistical Analysis

Data were analyzed according to a completely randomized block design with seven treatments. A Shapiro–Wilks normality test was carried out on the base data. The outliers were checked and excluded from the statistical analysis. Outliers were defined as values being more than twice the standard deviation from the mean. Levene’s test was used to examine group homogeneity among treatment groups. A one-way ANOVA was applied to performance data and other results, with the effect of treatment as the main factor of variation in body weight at different ages, FI and FCR, and the number of white blood cells, as well as morphometry parameters. To determine statistical significance (*p* < 0.05), Tukey’s post-hoc test was applied to check the differences between treatment groups. Frequency distributions were calculated for the histological injuries of liver tissue samples along with a Kurksal–Wallis test to determine the ordinal scale of the injuries.

## 3. Results

### 3.1. Growth Performance and Feed Efficiency

**Figure 2:**
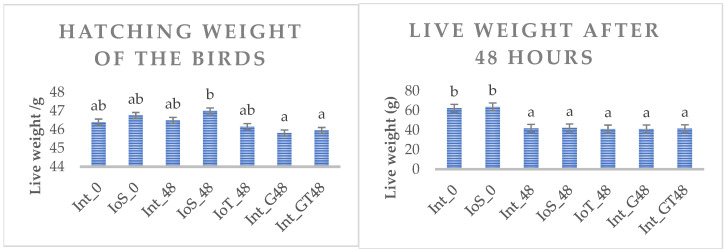
Hatching weight and live weight at 48 h post-hatch of broilers in different experimental treatment groups. Bars represent treatment means and SD. ^a,b^ different letters indicate a significant difference (*p* < 0.05).

Hatchability (hatched chicken/total number of eggs) was in the range of 0.74–0.79 in eggs having no intervention (intact eggs), and 0.77–0.78 in groups applied physiological saline solution and 0.81 when in ovo threonine supplementation was applied at day 17 of the incubation. The live weight of birds in different experimental treatments at hatch and 48 h later is shown in Figure 2. The ANOVA confirmed a significant difference in hatching weight, as birds in one of the in ovo saline-treated groups assigned to the IoS_48 group were 3% heavier than all others (*p* < 0.05). Live weight on the third day of the trial was much higher (34.3%) in birds fed post-hatch (Int_0 and IoS_0 vs. other groups) immediately.

The effect of dietary treatments that include early threonine supplementation on LW, FI, FCR, and ADG of broilers is shown in Table 4. Birds with immediate feed access reached 240 g on average by day 10, while birds with a 48 h delay in access to the first solid feed had more than 20% lower body weight (194 g on average). At the end of the starter phase, eggs supplemented with in ovo saline (IoS_0) had 5.1% higher LW than the intact group (Int_0), while treatments with 48 h of delayed feeding did not differ from each other. LW at day 21 was the highest in birds fed immediately post-hatch and differed significantly from the different groups (Int_0 and IoS_0 differed from 11.29% of the others, *p* < 0.05). The lowest LW was recorded in the Int_48 group, and of the delayed groups, the in ovo Thr-treated birds had the highest body weight at the end of the grower phase. At the end of the trial, on day 35, the immediate feed birds still had the highest LW (6% higher than others). Compared to their immediate feed counterparts, there was no statistically confirmed difference between the LW of the delayed and in ovo Thr or hydrogel supplemented birds. Group IoT_48 did not significantly differ either from the IoS_48 and Int_48GT or from the Int_48, which had the lowest final LW.

The FI of the birds showed significant differences at all the examined time intervals; however, the advantage of groups Int_0 and IoS_0 remained constant. Statistics confirmed a significant treatment effect on FCR in the starter, grower, and finisher phases. In ovo Thr-supplemented birds had 2.5% better feed utilization in the starter phase than birds who received their diet immediately post-hatch and were treated with in ovo saline (IoS_0, *p* = 0.04). The average daily gain was significantly affected by dietary treatments in all phases and the whole trial. In the starter phase, the 48 h delay in FI resulted in a 24% lower growth rate (Int_0 and IoS_0 vs. others, *p* < 0.05). In the grower and finisher phases, the advantage of the immediate feed birds was obvious, while supplementation of in ovo Thr and the hydrogel with or without Thr enrichment could compensate at least partly to the loss caused by the late feed access.

### 3.2. Intestinal Morphometry

Table 5, Table 6 and Table 7 present the gut morphology results affected by dietary treatments at hatch, 48 h post-hatch, and 21 days of age, respectively.

The intestinal morphological results on villus height measured at hatch (Table 5) show a significant difference in the duodenum between the IoS_0 and Int_48 groups (25%), in the ileum between the Int_0 and IoS_0 groups and the IoS_48 and Int_G48 groups (23%), and between the IoS_48 and IoT_48 groups and the Int_GT48 (30%) group in the colon region. Crypt depth was affected in the duodenum and the colon. The IoS_48 group had the lowest crypt depth, as it differed significantly from Int_0, IoS_0, Int_48, and IoT_48 groups in the duodenum (11% lower crypt depth than the other groups). In the architecture of the colon, birds assigned to in ovo saline (IoS_0 and IoS_48) and Int_GT48 group showed the lowest crypt depth, while that of birds in Int_48 and IoT_48 treatments had the highest.

The villus height was affected by treatments in two examined intestinal sections on day 3 (48 h post-hatch) (Table 6). It was the highest in the Int_0 group and the lowest in the IoS_0 group both in the duodenum and colon. Crypt depth was statistically the same in all groups. VH/CD ratio was affected only in the colon, showing the lowest rate in the Int_48 group differed significantly by 29% from that of birds in Int_0, IoS_0, Int_G48, and Int_GT48.

Significant differences were found on day 21 regarding villus height in the duodenum and the ileum, but not in the colon (Table 7). A statistically confirmed, 17% difference was obtained in duodenal villus height between Int_48 and Int_G48 birds, while all others did not differ. The ileal villi were the longest in birds assigned to IoT_48 treatments, and differed from almost all other groups, except for birds in IoS_0. Crypt depth was not affected by dietary treatments. Villus height/crypt depth ratio was different between Int_0 and Int_G48 (32%), and between IoT and Int_GT48 in the duodenum and ileum (29%), respectively.

### 3.3. Liver Histology and Blood Cell Results

Results of the liver histology demonstrate that most of the alterations regarding the heterophil granulocyte infiltration within the periportal regions occurred right after hatch and remained constant by day 21 (Table 8). Interestingly, it showed the worst results in the in ovo treated groups by day 3. From day 3, another pathological change, mononuclear infiltration, appeared in the liver, mostly in the periportal areas, but sometimes as multifocal lesions scattered in the parenchyma. This lymphohistiocytic invasion increased and gradually became predominant by day 21 in almost all groups. In addition, hepatocytes showed moderate to marked lipid accumulation in every section. This hepatic lipidosis gradually reduced from day 1 to day 21 and completely disappeared in most of the groups. Only in groups Int_G48 and Int_GT48 was a mild lipidosis detected on day 21, which remained as a faint vacuolization in group IoS_48 by this time.

Due to a technical failure, determination of leukocyte ratio was not successful in Int_0 birds and the data of that treatment were discarded from statistical analysis. With blood smear evaluation, moderate lymphocyte depletion and a mild increase in monocyte numbers were detected in all groups and ages (Table 9). Heterophil and eosinophil granulocyte numbers did not show remarkable alterations to dietary treatments (*p* > 0.05).

## 4. Discussion

Our study aimed to evaluate the efficiency of different early feeding methods by revealing some physiological changes that might be induced by post-hatch feed deprivation. We investigated whether the provided early threonine supplementation, either before or after hatch, could alleviate the negative impact of delay in solid feed access. In ovo feeding was first reported several decades ago, yet it is still not a common practice in hatcheries. However, an increasing number of studies have evaluated the effect of specific nutrient supplementation to the poultry embryo on the growth performance of meat-type poultry and reveal a course of action for the supplementation.

In the present study, in ovo intervention did not reduce the hatchability of the eggs. Some studies have reported a lower hatchability rate when in ovo feeding [36,37,38]; however, it seems that not the intervention itself but the circumstances, like the amount, concentration, and osmolarity of the injected solution, etc., were responsible for the lower hatch rate in different studies [39]. Statistically confirmed difference was observed between the hatching weight of IoS_48 and that of the other groups; however, that result is ambiguous. The in ovo saline solution was used as a control, per se, in the in ovo intervention. The treatments IoS_0 and IoS_48 can be considered as identical groups at the time of hatching, since in their case, in ovo manipulation took place on day 17 of incubation, and at hatch, even the intake of feed did not cause any difference, since the Int_0 birds were settled after the weighing. Therefore, we can conclude that eggs in groups of IoS_0 and IoS_48 were treated equally. In agreement with our results, Kadam et al. [40] confirmed in a meta-analysis that the effect of physiological saline is non-consequential and the treated birds do not always have higher weight at hatch. The beneficial effects of in ovo feeding on hatching weight are majorly shown when carbohydrates are used as nutrient supplementation [8,10,41]. In line with other results [40,42], threonine supplementation on day 17 of incubation did not shift hatching weight in the present study. It is well documented that birds at hatching need energy and thus, carbohydrates can be promptly used as a fuel to compensate for the energy deficit at hatch. In the case of an amino acid supplementation, the aim was not to support the energy status, but to provide nutrients as building blocks for specific proteins like epithelia or functional proteins involved in defense mechanisms. We hypothesized that early Thr supplementation provided either during late embryo development or in the post-hatch period might contribute to better gut tissue development and result in a better growth rate and more efficient birds. That has been at least partly confirmed. Live weight and the average daily gain of broilers were supported by early threonine supplementation when the birds had a 48 h delay to solid feed access. However, it has to be admitted that the performance may still be slightly compromised, and the feed deprivation in the first two days of life may be only minimally compensated by either of the early methods of Thr supplementation. Immediate post-hatch feeding is an ideal situation, but it is hard to carry out in practice. Birds get their first feed within a 36–72 h delay very often. Therefore, in our study, the immediate feed group represented the genetic potential of the birds. In a recent work by Alabi et al. [42], early (within 2 or 24 h) post-hatch feeding compared to a 48-h delay to the first feed improved the growth performance of broilers, most likely due to improved hormone secretion (T3, T4, and IGF-1), and also by enhancing the intestinal health and modulating the microbiota, especially at day 21. Results of de Jong et al. [3] also confirmed that even a 24–36 h delay in post-hatch feed access reduces the body weight of chicks to a statistically verifiable extent. It has to be noted, however, that the longer the fattening period (e.g., 50 days), the more ability the birds have to compensate for the early perturbation.

The provision of gel supplements is the most widely used in practice among early feeding methods. Several studies report the beneficial effects of hydrogels, such as hydrating the day-old birds or adding probiotic benefits [43,44]. Even though it is a rich supplement with high moisture content, hydrogel could not compensate for the two-day feed deprivation, as the body weight of birds was lower on day 3 than on day 1 of the trial. This result emphasizes that the energy supply from the yolk sac is not enough even for maintenance purposes in intensive genotypes. As reviewed by Al-Huwaizi [45], the yolk sac nutrients––particularly fatty acids––can be more efficiently utilized if birds receive solid feed post-hatch. Among delayed feed birds, the early access to hydrogel, particularly with Thr enrichment, resulted in the highest body weight at the end of the experiment numerically, but not in the starter and grower phases.

In the first three weeks, the in ovo Thr supplementation improved the growth performance, like ADG and FCR, of birds compared to their non-supplemented counterparts. It has been repeatedly confirmed that in ovo supplementation of Thr alone or in combination with carbohydrates or other amino acids like arginine can improve the growth rate of broilers [46,47,48,49,50]. Those positive results were almost completely explained by the improvements of the morphology of the gut.

It is well-documented that dietary threonine has a key role in intestinal development from both structural and functional points of view [51,52]. Thr, being an essential amino acid, plays a vital role in the maintenance of intestinal barrier integrity and mucin synthesis [52,53]. It is the most abundant essential amino acid in the endogenous protein secreted in the intestine, particularly in the mucins, in which Thr represents 16% of total amino acids [54]. In conventional feeding trials, it has been confirmed that insufficient threonine supplementation has a negative effect on the morphological state of the intestine [55,56]. Moreover, providing Thr supplementation above the recommended level resulted in higher villus length, lower crypt depth, and improved VH/CD ratio in broilers, compared to the control group [53]. In contrast with Ospina-Rojas et al. [57], however, who did not find a statistically reliable difference in gut morphology when feeding diets with an increased threonine content.

Earlier studies have reported that, under commercial conditions, a level of Thr that exceeds the current NRC recommendation [58] is required to achieve maximum immune function and health status for poultry [59,60,61]. Thus, it is an interesting issue whether a short-term, but targeted Thr supplementation has benefits for broilers.

The intestinal morphological results measured at hatch can hardly be explained by consequent dietary treatments. Overall, we could not confirm any positive effect of the provided threonine supplementation during the embryonic stage on brush border development, at hatch. However, it seems that early Thr supplementation may have a positive effect on the architecture of the intestinal epithelium, particularly on the villus height/crypt depth ratio, which is correlated with the absorptive surface of the gut later on. In agreement with other studies [43,62,63,64], our results show that post-hatch feed deprivation compromises the ideal trajectory of gut tissue maturation and there is a significant difference between brush border architecture when birds fed immediately or after a 48 h delay post-hatch. Moreover, data from Proszkowiec-Weglarz et al. [65] suggest that delay in feeding may indirectly affect the gut barrier function of the small intestine as well as possibly reduce the absorption and utilization of nutrients such as carbohydrates in the intestinal tract of broilers. Those negative effects can be at least partly compensated if gel supplements or early Thr supplementation is provided, as confirmed by our results. In line with these findings, numerous studies [48,66,67] reported higher villi and more intensive mucin secretion in birds who received in ovo Thr.

Hepatic tissue plays a key role in lipid, protein, carbohydrate and vitamin metabolism, and other metabolic, homeostatic, and immune functions. Feed restriction at the early postnatal stage may have a long-term effect on the lipid metabolism of broiler chickens through the alteration of hepatic enzyme activity (as reviewed by Zaefarian et al. [68]). Meanwhile, as one of the most dynamic organs, the liver contributes highly to the adaptation and compensation ability of birds. Periportal heterophilic infiltration in day-old chickens and the following mononuclear invasion at the same histological sites might indicate acute inflammatory response in the young birds, that then developed into a chronic process in the older animals [69]. The origin of acute inflammation remains unknown, although the nature of the lesions may indicate microbial infection before or on the occasion of hatching [70]. As no significant connections were detected between the pathological alterations and the different groups, we can conclude that in ovo treatment neither could induce the histological changes nor had any impact on their further behavior. Hepatic lipidosis in young poultry is generally a natural process that can be connected to lipid transportation from the yolk sac and the slower lipid metabolism in day-old chickens. Usually, these processes induce only mild fat accumulation in a few days after hatching [65], although more severe and long-lasting lesions were observed in our study. The inflammation resulting in hepatocellular damage could be in the background of prolonged and more prominent lipidosis.

The reduced lymphocyte counts together with increased monocyte numbers in blood work means the so-called stress leukogram both in mammal and bird species is the result of glucocorticoid effects on blood cells and bone marrow [47]. Our findings indicate that chickens had some degree of stress during their development, although its origin was probably not the nature of experimental treatments, because stress leukogram patterns could be observed in all groups and ages.

Early feeding techniques aim to support the intensive growth potential of poultry. To date, post-hatch feeding solutions are commonly used in practice. Although in ovo vaccination techniques are widespread, in ovo feeding to nourish embryos before hatching is not widely adopted, despite numerous research findings confirming its ability to enhance chick performance and health during the growing and fattening periods. In ovo feeding addresses the nutrient gap during the lag period (from hatch to the first feed). While combining supplements may offer additional benefits, understanding the specific contribution of each ingredient is crucial. Some bioactive compounds delivered in ovo have shown effects similar to antibiotic growth promoters; however, long-term trials are needed to evaluate their effectiveness throughout the post-hatch period [71,72]. There is a common agreement that for wider commercial adoption, standardized injection procedures are essential to ensure consistent and reproducible results.

## 5. Conclusions

Our results confirmed that the best performance was achieved in birds without delay in feed access. Provision of Thr at early ages both in ovo and through hydrogel could at least partly compensate for the loss attributed to the 48 h delay in feed access post-hatch. The higher compensation ability can be explained by the better architecture of the gut tissue, since in ovo Thr feeding supported growth and resulted in favorable villus height/crypt depth ratio in the first three weeks. As a conclusion of our research, specific early nutrition techniques, such as in ovo feeding of Threonine, can be recommended when post-hatch feed deprivation is anticipated. However, further studies are needed to clarify the reasons behind the loss of the performance advantage observed with in ovo Thr supplementation by the end of the study. Based on performance outcomes, hydrogel fortified with or without Thr is recommended as a practical intervention for broilers experiencing delayed access to feed.

## Figures and Tables

**Table 1:** Temperature and CO_2_ level during incubation.

Hatching Day		°C	Humidity %	CO_2_ Concentration %
1	Incubation	37.9	70	0.60
2		37.9	70	0.60
3		37.9	70	0.60
4		37.9	62	0.60
5		37.9	56	0.60
6		37.9	56	0.60
7		37.8	55	0.60
8		37.8	56	0.60
9		37.6	55	0.60
10	Candling	37.6	56	0.60
11		37.5	56	0.35
12		37.5	56	0.35
13		37.4	55	0.35
14		37.3	55	0.35
15		37.3	55	0.35
16		37.2	56	0.35
17	Candling, in ovo intervention, placing into the incubator	37.1	56	0.35
18		37.0/36.7	56	0.35/0.60
19		36.7	55	0.60
20		36.5	56	0.60
21		36.2	56	0.60
22		36.2/35.8	62	0.35

**Table 2:** Definition of treatment groups in the study.

Treatment Code	Feed Access	Early Nutrition Method	Number of Eggs
Int_0	Immediate	-	160
IoS_0		in ovo, saline	160
Int_48	48 h delayed solid feed access	-	160
IoS_48		in ovo, saline	160
IoT_48		in ovo Thr	160
Int_G48		Hydrogel	160
Int_GT48		Hydrogel + Thr	160

**Table 3:** Analyzed feed composition in the three feeding phases.

Ingredients	Starter (1–10)	Grower (11–21)	Finisher (22–35)
Corn (grain)	551	577	601
Corn gluten (60%)	32	32	32
Sunflower meal	53.5	53.5	75
Soybean meal (CP 44.2%)	262	230	175
Fat, vegetable	44.7	55	67.00
Monocalcium phosphate	18.7	17.5	15
Limestone	15	13.5	12.2
NaCl	2.7	2.7	2.7
L-Lysin HCl	5.2	4.6	4.3
DL-Methionine	4.5	3.9	3.2
L-Threonine	2.6	2.3	1.8
Premix ^1^	5.00	5.00	5.00
Total	1000.00	1000.00	1000.00
Nutrient content (g/kg)			
AMEn (MJ/kg)	12.5	12.9	13.4
DM %	90	91.3	91.1
Crude protein	204.2	190.7	174.9
Crude fat	71.87	82.3	94.4
Crude fiber	41.5	41.1	44.8
Lysine *	13.5	12.1	10.8
Methionine + Cystine *	10.8	9.9	9.0
Threonine *	9.7	8,8	7.8
Tryptophane *	2.4	2.3	1.7
Ca	9.6	8.7	7.8
P_available_	4.7	4.5	3.9
Na	1.7	1.7	1.7

^1^ Premix feed contents per kilogram: Zn: 22,032 mg, Cu: 3200 mg, Fe: 16,020 mg, Mn: 21,948 mg, I: 300 mg, Se: 70 mg, Co: 20 mg, Vit. A: 3,240,000 IU, Vit. D3: 810,000 IU, Vit. E: 20,800 mg, Vit K3: 810 mg, Vit. B1: 810 mg, Vit. B2: 1890 mg, Vit. B3: 10,800 mg, Vit. B5: 3240 mg, Vit. B6: 1350 mg, Vit B12: 6.8 mg, Folic acid: 270 mg, Biotin: 32 mg. * Calculated values.

**Table 4:** Effect of dietary treatments on feed intake (FI), feed conversion ratio (FCR), and average daily gain (ADG) in different phases and the whole experiment.

	Int_0	IoS_0	Int_48	IoS_48	IoT_48	Int_G48	Int_GT48	RMSE	*p*-Value
Body weight (g)									
d10	233 ^a^	245 ^b^	191 ^c^	193 ^c^	196 ^c^	194 ^c^	192 ^c^	25.8	<0.0001
d21	855 ^ab^	882 ^a^	766 ^d^	782 ^cd^	809 ^bc^	777 ^cd^	785 ^cd^	95.3	<0.0001
d35	2218 ^a^	2238 ^a^	2072 ^b^	2113 ^ab^	2086 ^b^	2096 ^ab^	2100 ^ab^	257.4	<0.0001
Feed intake (kg/day/pen)									
d1-10	23.2 ^a^	24.0 ^a^	17.9 ^b^	17.7 ^b^	17.8 ^b^	17.1 ^b^	17.9 ^b^	1.72	<0.0001
d11-21	72.8 ^ab^	75.6 ^a^	66.8 ^c^	66.7 ^c^	69.1 ^bc^	67.3 ^c^	66.8 ^c^	1.36	<0.0001
d22-35	145.6 ^a^	144.4 ^a^	136.9 ^b^	138.6 ^ab^	137.6 ^b^	138.6 ^ab^	140.1 ^ab^	6.22	0.04
d1-35	85.35 ^a^	86.3 ^a^	78.3 ^b^	78.2 ^b^	79.3 ^b^	78.9 ^b^	79.1 ^b^	3.52	<0.0001
Feed conversion ratio (kg feed/kg gain)									
d1-10	1.24 ^a^	1.21 ^ab^	1.24 ^a^	1.21 ^ab^	1.18 ^b^	1.15 ^b^	1.23 ^ab^	0.13	0.04
d11-21	1.28 ^ab^	1.30 ^a^	1.28 ^ab^	1.24 ^ab^	1.24 ^b^	1.26 ^ab^	1.23 ^b^	0.77	0.001
d22-35	1.49 ^b^	1.48 ^b^	1.45 ^ab^	1.45 ^a^	1.50 ^b^	1.46 ^a^	1.48 ^b^	0.13	0.03
d1-35	1.37 ^ab^	1.37 ^ab^	1.35 ^b^	1.32 ^bc^	1.36 ^ac^	1.34 ^c^	1.34 ^c^	0.13	0.043
Average daily gain (g/d)									
d1-10	18.6 ^b^	19.8 ^a^	14.4 ^c^	14.6 ^c^	15.0 ^b^	14.8 ^b^	14.5 ^b^	2.57	<0.0001
d11-21	56.5 ^ab^	57.9 ^a^	52.1 ^c^	53.5 ^bc^	55.6 ^ab^	53.0 ^bc^	53.9 ^bc^	7.1	<0.0001
d22-35	97.5	97.1	93.8	95.0	91.2	94.5	94.2	14.28	0.13
d1-35	62.0 ^a^	62.6 ^a^	57.8 ^b^	59 ^ab^	58.3 ^b^	58.5 ^ab^	58.7 ^ab^	7.19	<0.0001

^a,b,c^ means in the same row with different letters statistically differ (*p* < 0.05).

**Table 5:** Effect of dietary treatments on villus height, crypt depth, and villus height/crypt depth ratio in the duodenum, ileum, and colon (D, I, and colon, respectively) at hatch.

	Int_0	IoS_0	Int_48	IoS_48	IoT_48	Int_G48	Int_GT48	*p*-Value
Villus height (µm)								
D	580.8 ^ab^	506.5 ^a^	673.2 ^b^	639.8 ^ab^	621.8 ^ab^	579.2 ^ab^	586.1 ^ab^	0.032
I	302.6 ^a^	295.4 ^a^	363.6 ^ab^	375.3 ^b^	358.7 ^ab^	400 ^b^	364.5 ^ab^	0.0011
C	318.3 ^ab^	377.8 ^a^	347.2 ^ab^	308.1 ^ab^	357.3 ^a^	310.5 ^ab^	258.2 ^b^	0.0055
Crypt depth (µm)								
D	111.3 ^a^	110.3 ^a^	109.5 ^a^	80.5 ^b^	115.4 ^a^	100.1 ^ab^	103.9 ^ab^	0.001
I	88.1	94.6	93.3	94.9	83.4	106.0	85.6	0.16
C	92.5 ^ab^	87.4 ^a^	104.5 ^b^	81.4 ^a^	110.5 ^b^	94.4 ^ab^	82.0 ^a^	0.0004
Villus height/Crypt depth ratio								
D	4.3 ^a^	4.6 ^a^	4.8 ^a^	8.2 ^b^	5.5 ^a^	5.1 ^a^	3.9 ^a^	<0.001
I	3.4 ^ab^	3.0 ^a^	3.8 ^ab^	4.0 ^ab^	3.8 ^ab^	3.8 ^ab^	4.4 ^b^	0.0082
C	4.0	4.2	3.5	4.0	5.5	3.4	3.3	0.28

^a,b^ means in the same row with different letters statistically differ (*p* < 0.05).

**Table 6:** Effect of dietary treatments on villus height, crypt depth, and villus height/crypt depth ratio in the duodenum, ileum, and colon (D, I, and colon, respectively) 48 h post-hatch.

	Int_0	IoS_0	Int_48	IoS_48	IoT_48	Int_G48	Int_GT48	*p*-Value
Villus height (µm)								
D	340.6 ^c^	214.9 ^a^	288.5 ^b^	240.8 ^b^	283.4 ^b^	321.6 ^bc^	250.0 ^ab^	<0.001
I	158.6	140.2	167.4	140.8	154.7	157.1	142.9	0.057
C	164.2 ^b^	126.4 ^a^	159.8 ^ab^	146.4 ^ab^	156.5 ^ab^	132.1 ^ab^	130.0 ^ab^	0.0085
Crypt depth (µm)								
D	36.3	29.2	35.6	34.0	36.5	31.8	35.6	0.43
I	32.5	30.3	33.7	29.6	32.6	33.6	32.8	0.14
C	29.9	25.3	30.3	29.1	28.3	26.5	26.7	0.059
Villus height/Crypt depth ratio								
D	7.7	8.1	8.1	7.6	7.8	9.2	7.2	0.28
I	4.8	4.5	4.8	4.8	4.8	4.7	4.6	0.96
C	5.7 ^b^	5.1 ^b^	3.7 ^a^	4.5 ^ab^	4.5 ^ab^	4.9 ^b^	4.9 ^b^	0.006

^a,b,c^ means in the same row with different letters statistically differ (*p* < 0.05).

**Table 7:** Effect of dietary treatments on villus height, crypt depth, and villus height/crypt depth ratio in the duodenum, ileum, and colon (D, I, and colon, respectively) at 21 days of age.

	Int_0	IoS_0	Int_48	IoS_48	IoT_48	Int_G48	Int_GT48	*p*-Value
Villus height (µm)								
D	768.9 ^ab^	748.8 ^ab^	830.9 ^a^	790.8 ^ab^	755.6 ^ab^	690.1 ^b^	765.8 ^ab^	0.04
I	210.6 ^a^	221.3 ^ab^	216.1 ^a^	199.3 ^a^	282.8 ^b^	218.7 ^a^	180.4 ^a^	0.0004
C	214.9	235.3	220.8	237.9	205.2	211.1	217.2	0.45
Crypt depth (µm)								
D	86.1	103.5	106.8	89.5	97.4	104.5	113.2	0.09
I	61.8	59.2	55.3	58.4	72.0	66.8	60.7	0.08
C	61.9	64.3	54.3	56.1	58.8	56.1	55.2	0.26
Villus height/Crypt depth ratio								
D	9.6 ^a^	7.8 ^ab^	8.1 ^ab^	9.1 ^ab^	8.2 ^ab^	6.6 ^b^	7.4 ^ab^	0.01
I	3.5 ^ab^	3.8 ^ab^	4.0 ^ab^	3.5 ^ab^	4.2 ^a^	3.3 ^ab^	3.0 ^b^	0.01
C	3.6	3.7	4.2	4.6	3.8	3.9	4.1	0.29

^a,b^ means in the same row with different letters statistically differ (*p* < 0.05).

**Table 8:** Effect of dietary treatment on heterophil granulocyte infiltration, vacuolization, mononuclear infiltration in the liver tissue, and lipid accumulation in liver cells at different time points of the experiment.

	Int_0	IoS_0	Int_48	IoS_48	IoT_48	Int_G48	Int_GT48	*p*-Value *
Heterophil granulocyte infiltration								
day 1	0.66 ^ab^	0.75 ^ab^	1.0 ^b^	0.58 ^a^	1.00 ^b^	1.00 ^b^	1.00 ^b^	0.01
day 3	0.41 ^ab^	1.00 ^b^	0.25 ^a^	0.57 ^ab^	1.00 ^b^	1.00 ^b^	0.75 ^ab^	0.0007
day 21	1.10 ^ab^	0.30 ^a^	0.58 ^a^	1.41 ^b^	1.66 ^b^	1.19 ^ab^	1.60 ^b^	<0.001
Vacuolization								
day 1	0	0	0	0	0	0	0	-
day 3	0.16 ^ab^	0 ^a^	0 ^a^	0.42 ^b^	0 ^a^	0 ^a^	0 ^a^	0.017
day 21	0 ^a^	0 ^a^	0 ^a^	0.28 ^b^	0 ^a^	0 ^a^	0 ^a^	0.007
Mononuclear infiltration								
day 1	0 ^a^	0 ^a^	0 ^a^	0.25 ^b^	0 ^a^	0 ^a^	0 ^a^	0.01
day 3	1.41 ^b^	0.33 ^a^	0 ^a^	0.71 ^ab^	0.50 ^a^	1.00 ^ab^	0 ^a^	0.008
day 21	1.77 ^ab^	2.00 ^b^	1.60 ^ab^	1.30 ^ab^	2.33 ^b^	1.33 ^a^	1.33 ^a^	<0.001
Lipid accumulation in liver cells								
day 1	1.00 ^a^	1.20 ^b^	1.00 ^a^	1.00 ^a^	1.00 ^a^	1.00 ^a^	1.00 ^a^	0.014
day 3	0.25 ^a^	1.66 ^c^	0.75 ^ab^	1.00 ^b^	1.00 ^b^	1.00 ^b^	1.00 ^b^	<0.001
day 21	0 ^a^	0 a	0 ^a^	0 a	0 ^a^	0.80 ^b^	0.40 ^ab^	<0.001

* represents Chi^2^ *p*-value. ^a,b,c^ means in the same row with different letters statistically differ (*p* < 0.05).

**Table 9:** Effect of dietary treatment on the leukocytes * in blood smear at hatch, 48 h post-hatch, and 21 days of age.

	Int_0	IoS_0	Int_48	IoS_48	IoT_48	Int_G48	Int_GT48	*p*-Value
At hatch								
HE	--	0.715	0.6	0.728	0.586	0.676	0.715	0.09
LYM	-	0.216	0.316	0.198	0.305	0.241	0.216	0.13
MON	-	0.019	0.036	0.026	0.025	0.016	0.019	0.10
EOS	-	0.048	0.048	0.047	0.068	0.067	0.048	0.86
48 h post-hatch								
HE	0.629	0.529	0.619	0.597	0.610	0.525	0.629	0.052
LYM	0.291	0.386	0.305	0.339	0.314	0.397	0.291	0.79
MON	0.035	0.023	0.028	0.025	0.034	0.028	0.035	0.90
EOS	0.045	0.038	0.048	0.039	0.042	0.050	0.045	0.05
21 days of age								
HE	0.314	0.323	0.296	0.346	0.317	0.357	0.314	0.33
LYM	0.578	0.579	0.613	0.570	0.571	0.563	0.578	0.82
MON	0.054	0.043	0.040	0.035	0.044	0.040	0.054	0.47
EOS	0.053	0.055	0.051	0.049	0.065	0.040	0.053	0.69

Leukocytes: HE = heterophils, LYM = lymphocytes, MON = monocytes, EOS = eosinophyls.

## Data Availability

The datasets presented in this article are not readily available because this data is part of an ongoing project due to technical due to technical limitations. Requests to access the datasets should be directed to halas.veronika@uni-mate.hu.

## Supplementary Materials

The following supporting information can be downloaded at: https://www.mdpi.com/article/10.3390/ani15030370/s1, File S1: Pathological injuries on the liver.
