# Supplementary material for: Impact of Threonine Supply in Early Ages on Gut Tissue Morphology, Liver Histology, and the Possible Changes in Leukocyte Numbers of Broilers
Source: Animals (Basel). 2025 Jan 27;15(3):370. doi: 10.3390/ani15030370 (PMC11815908; doi:10.3390/ani15030370)
Supplement: Supplementary file 1 [file animals-15-00370-s001.zip › Annex 1.pdf]

Annex 1

Representative images of liver histology in each treatment group

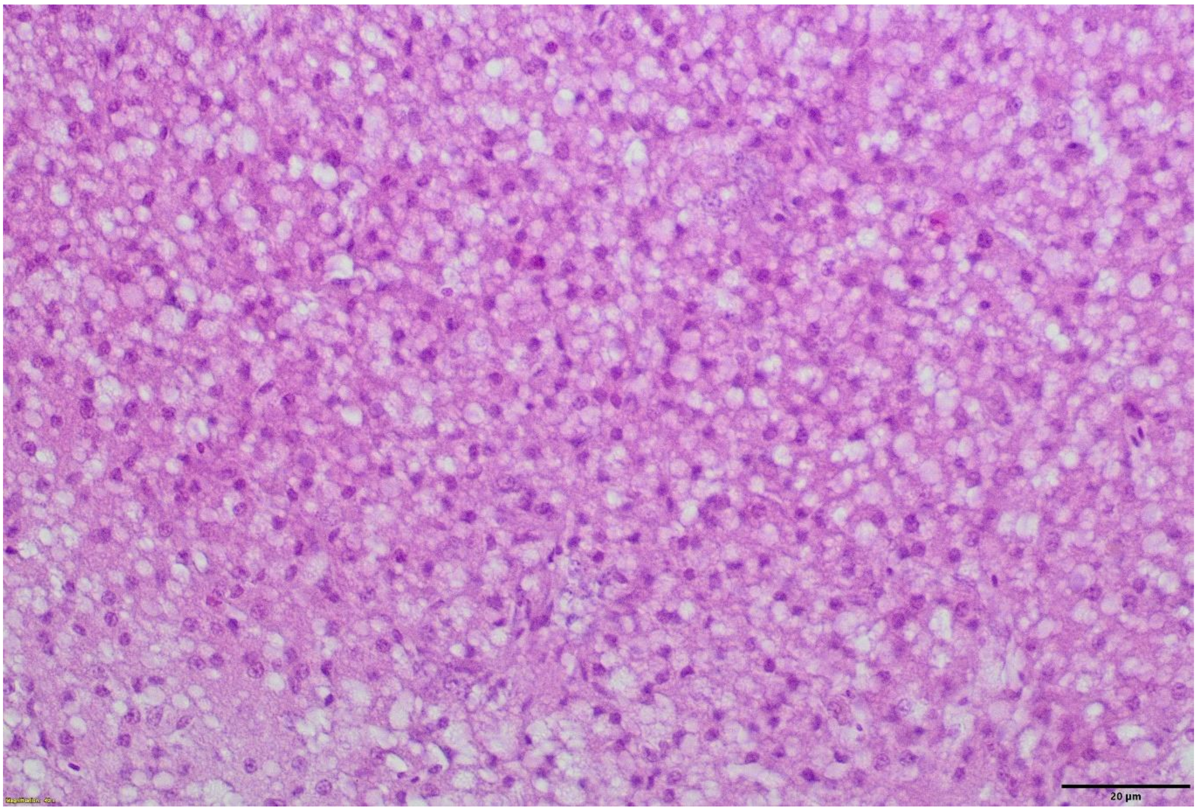

Int_0 day 1

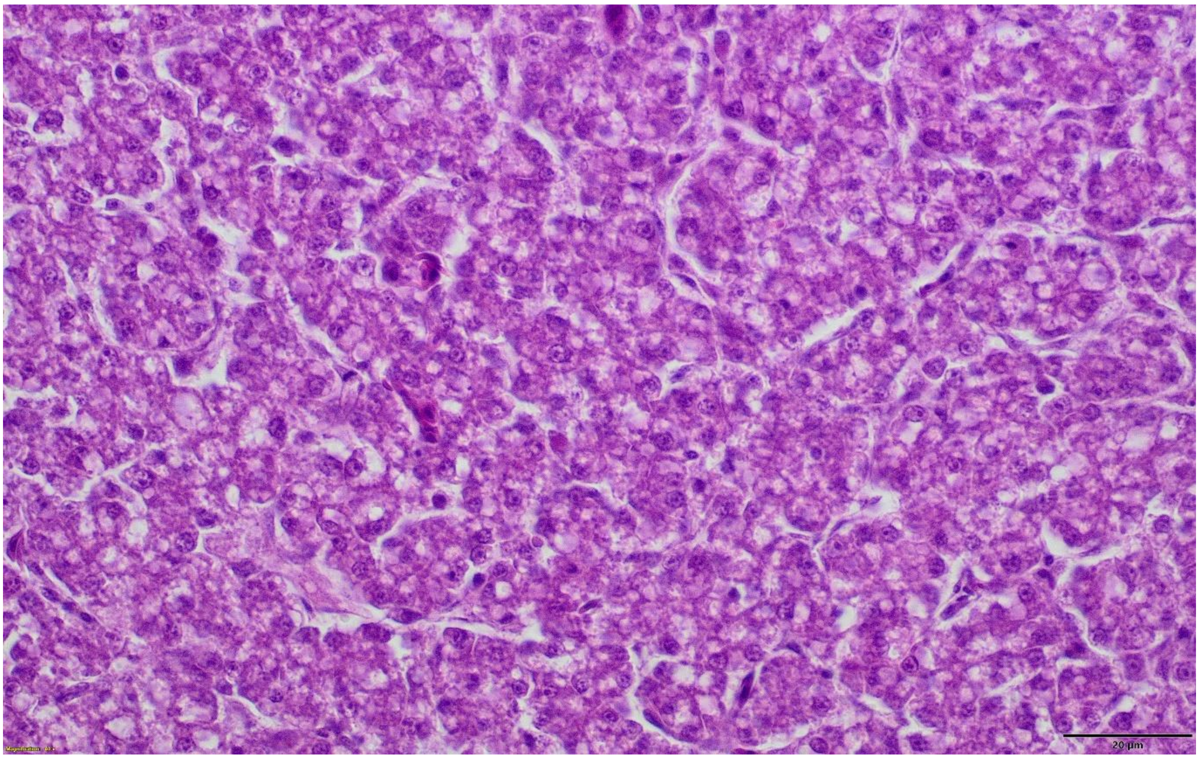

Int_0 day 3

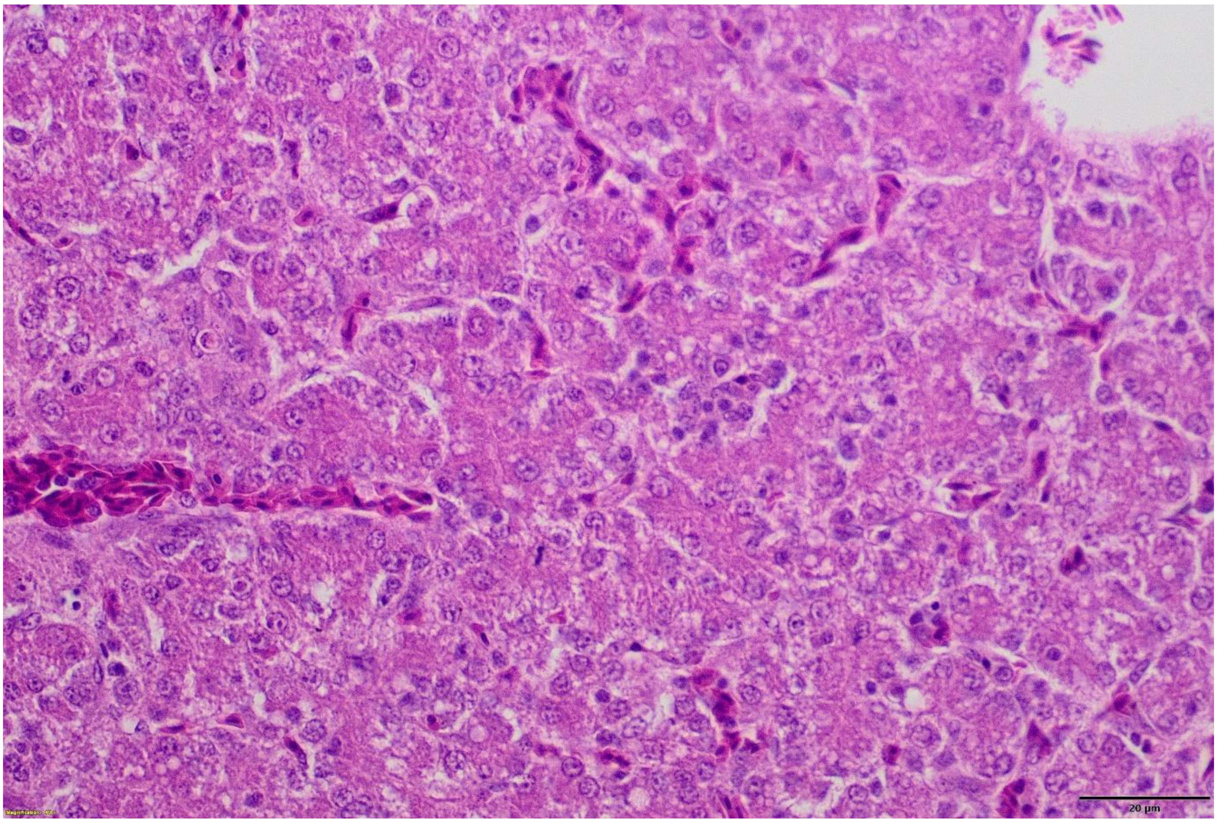

Int_0 day 21

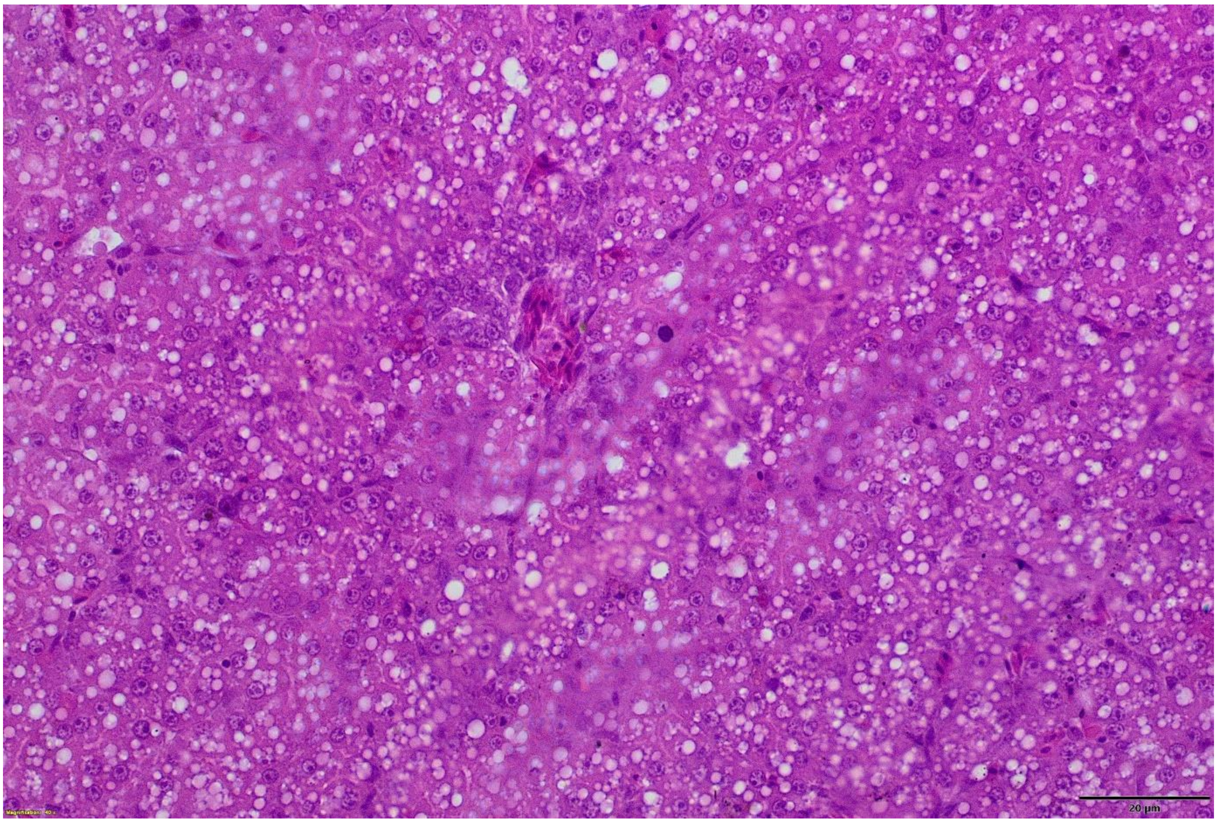

IoS_0 day 1

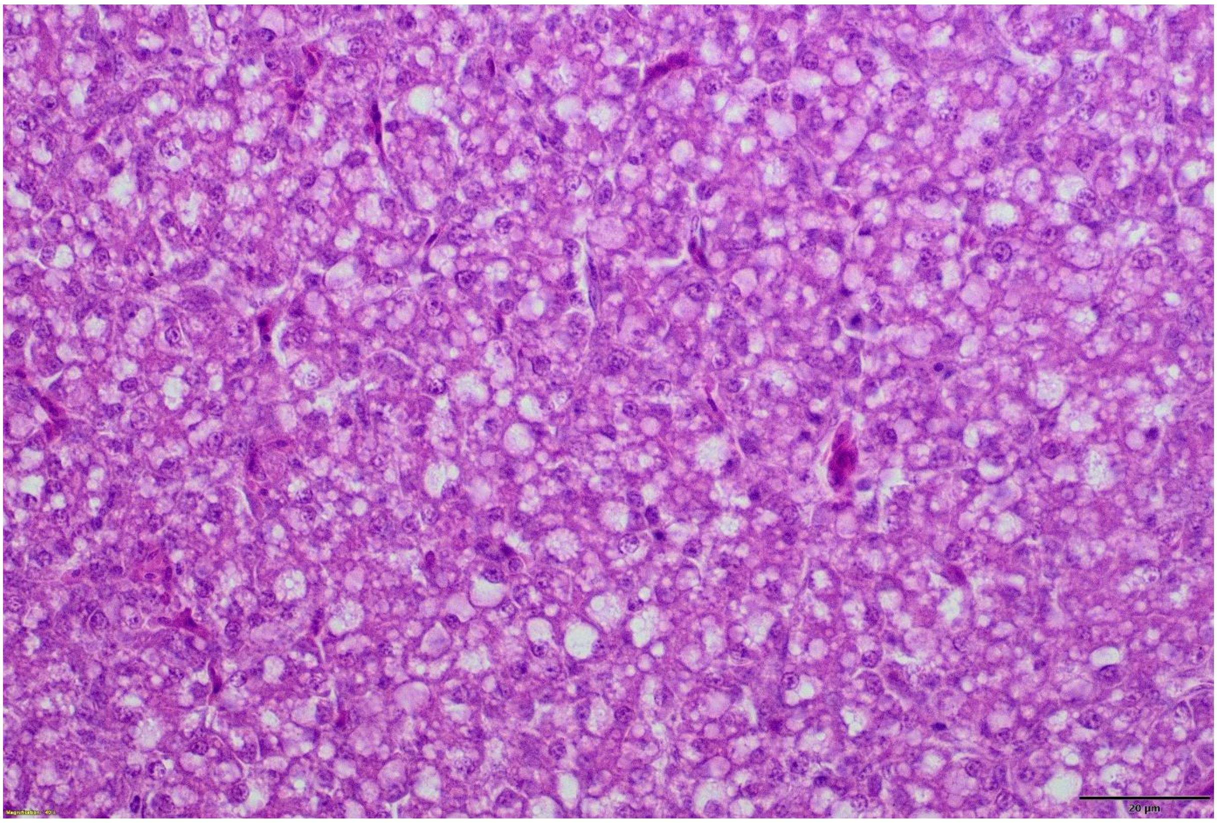

IoS_0 day 3

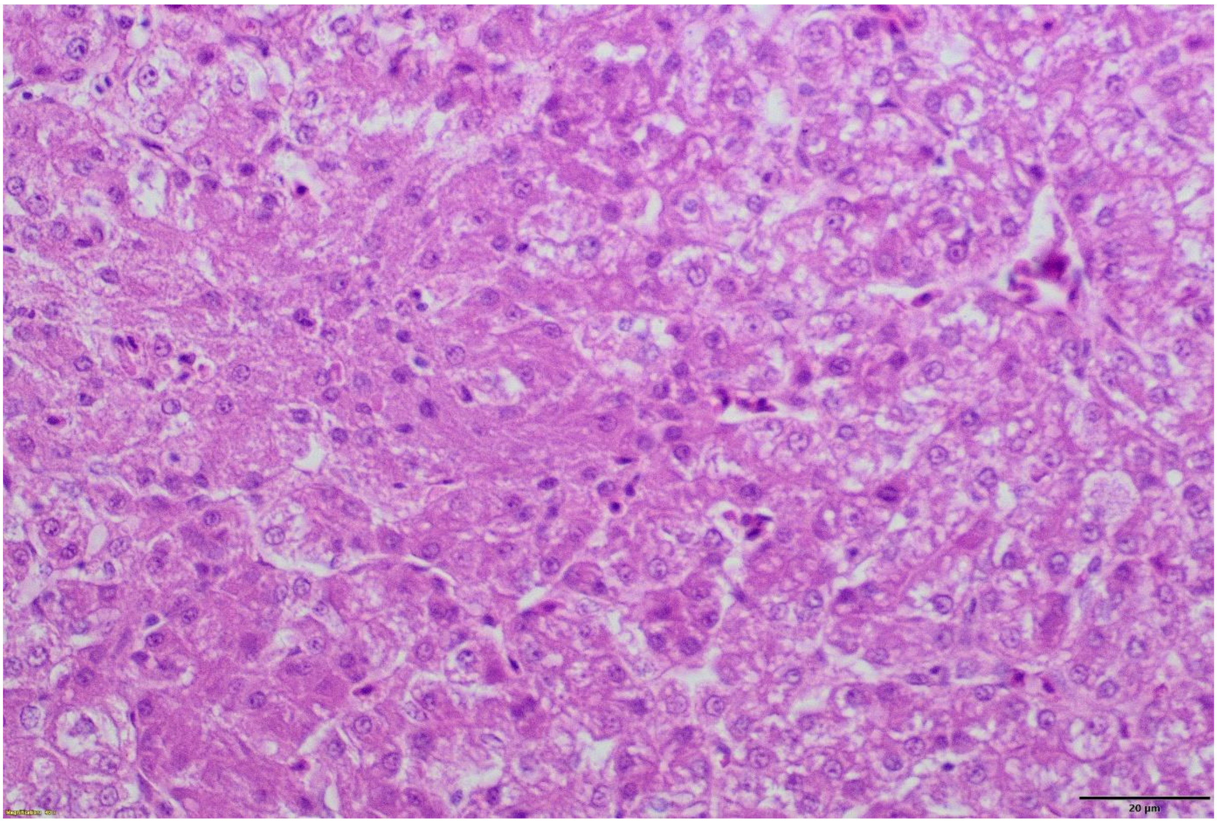

IoS_0 day 21

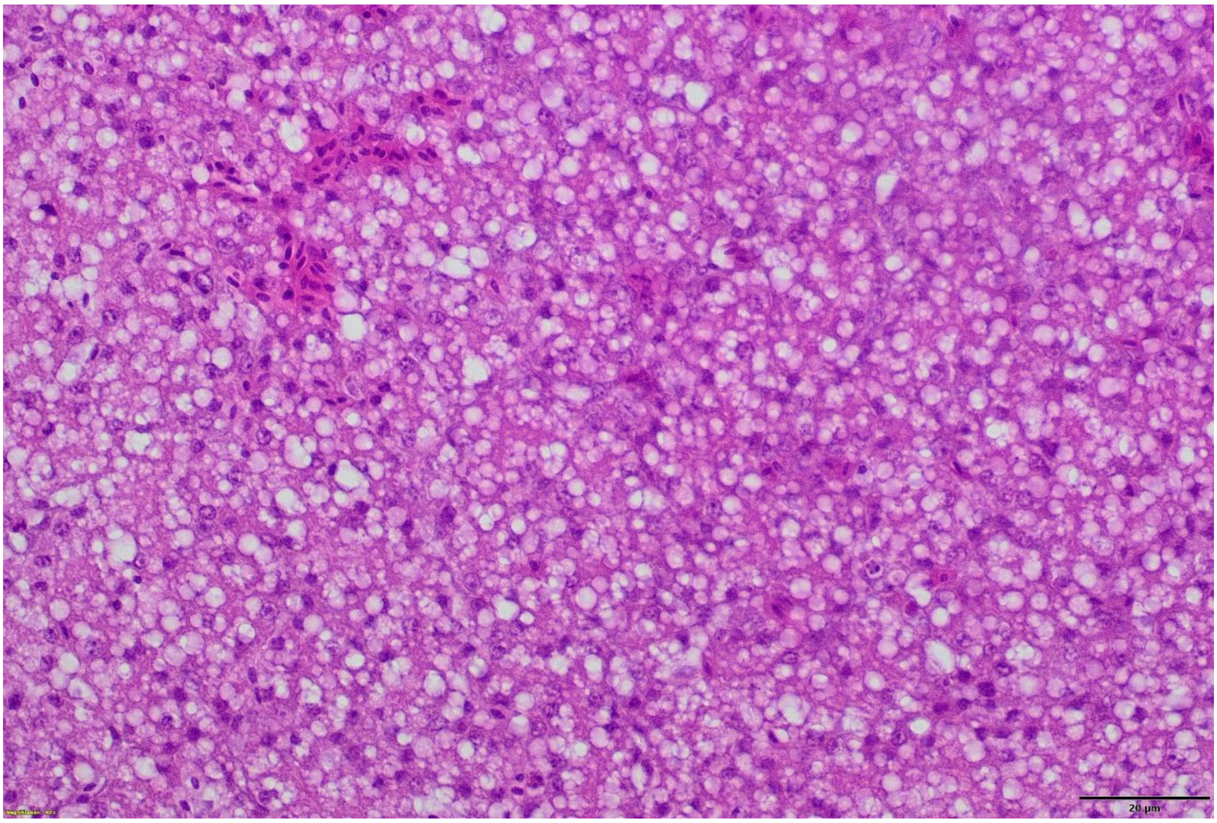

Int_48 day 1

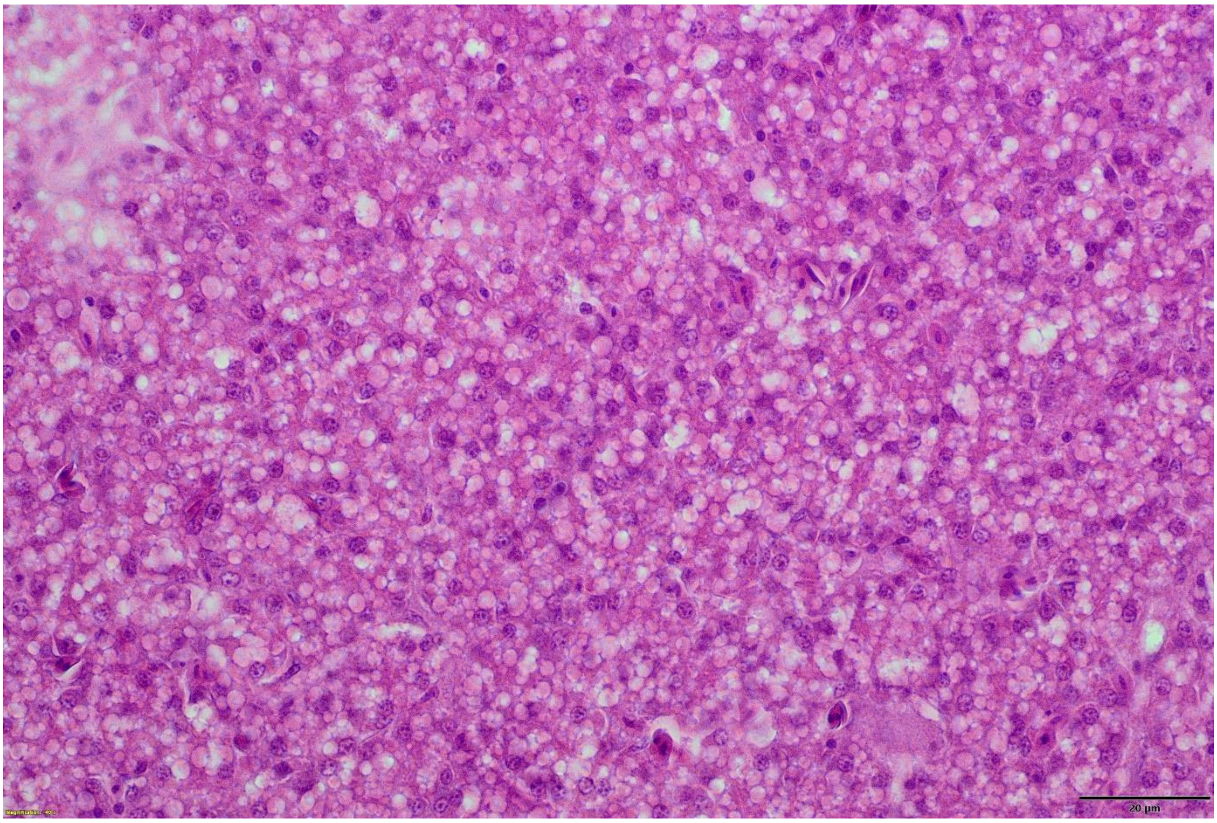

Int_48 day 3

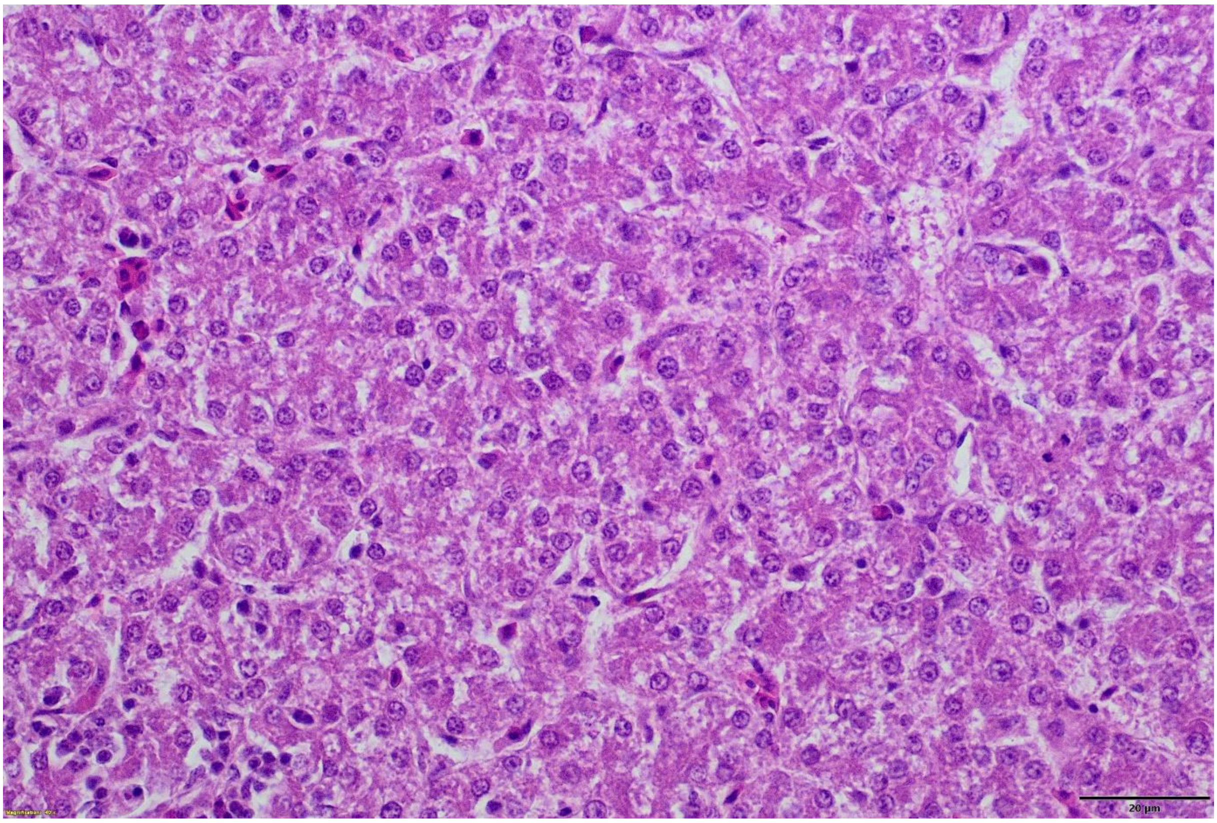

Int_48 day 21

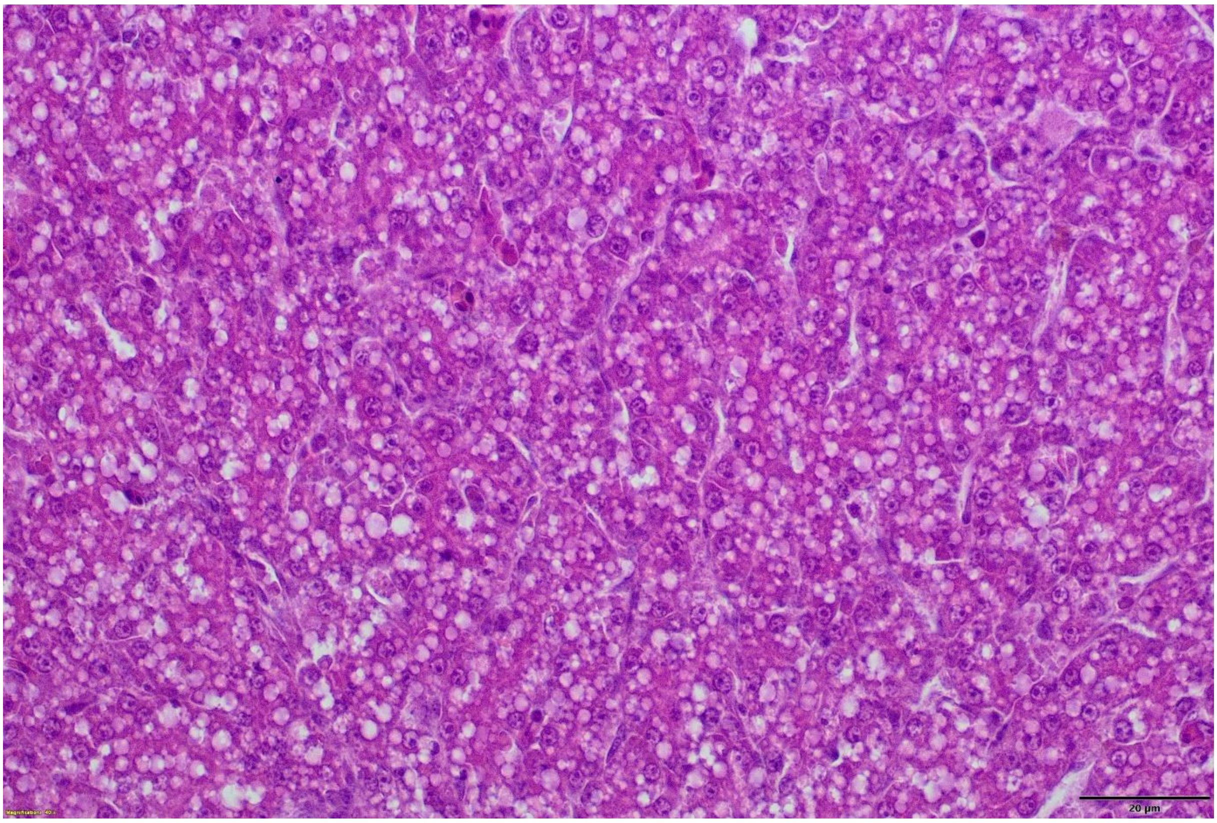

IoS_48 day 1

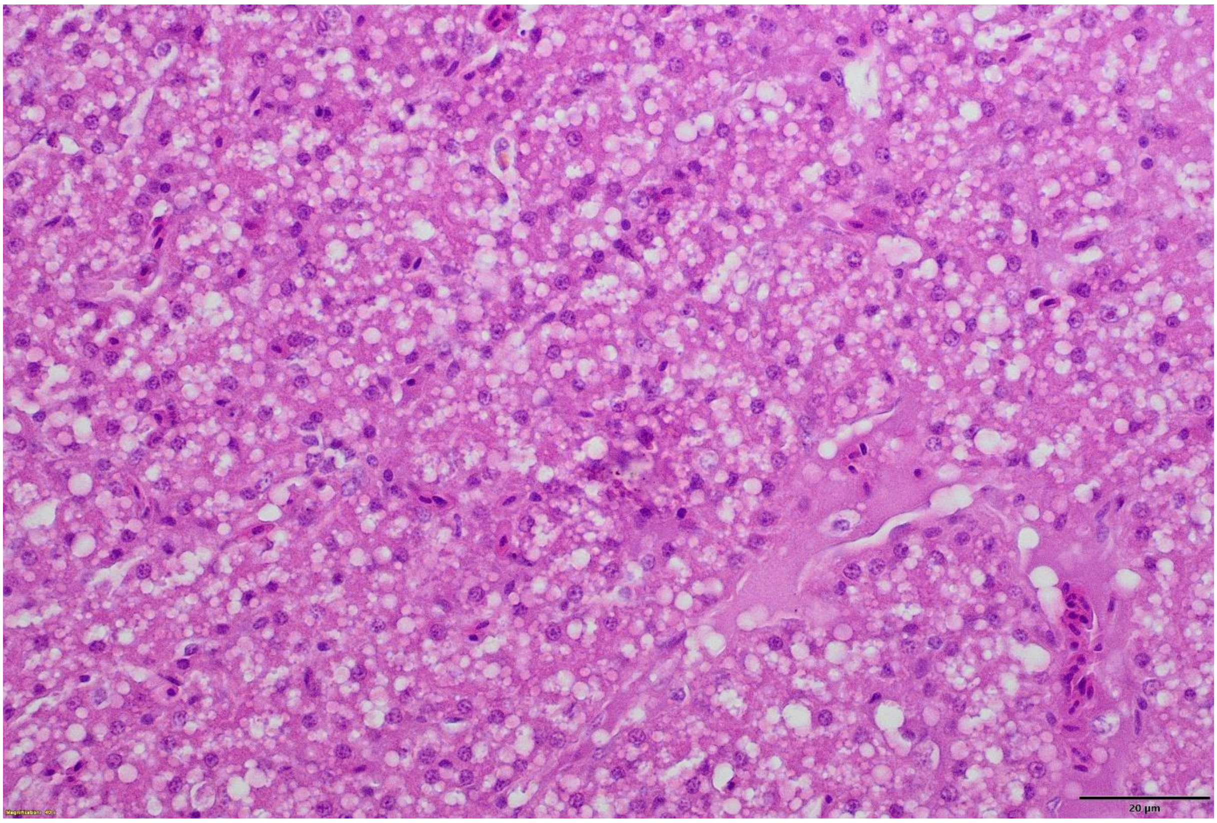

IoS_48 day 3

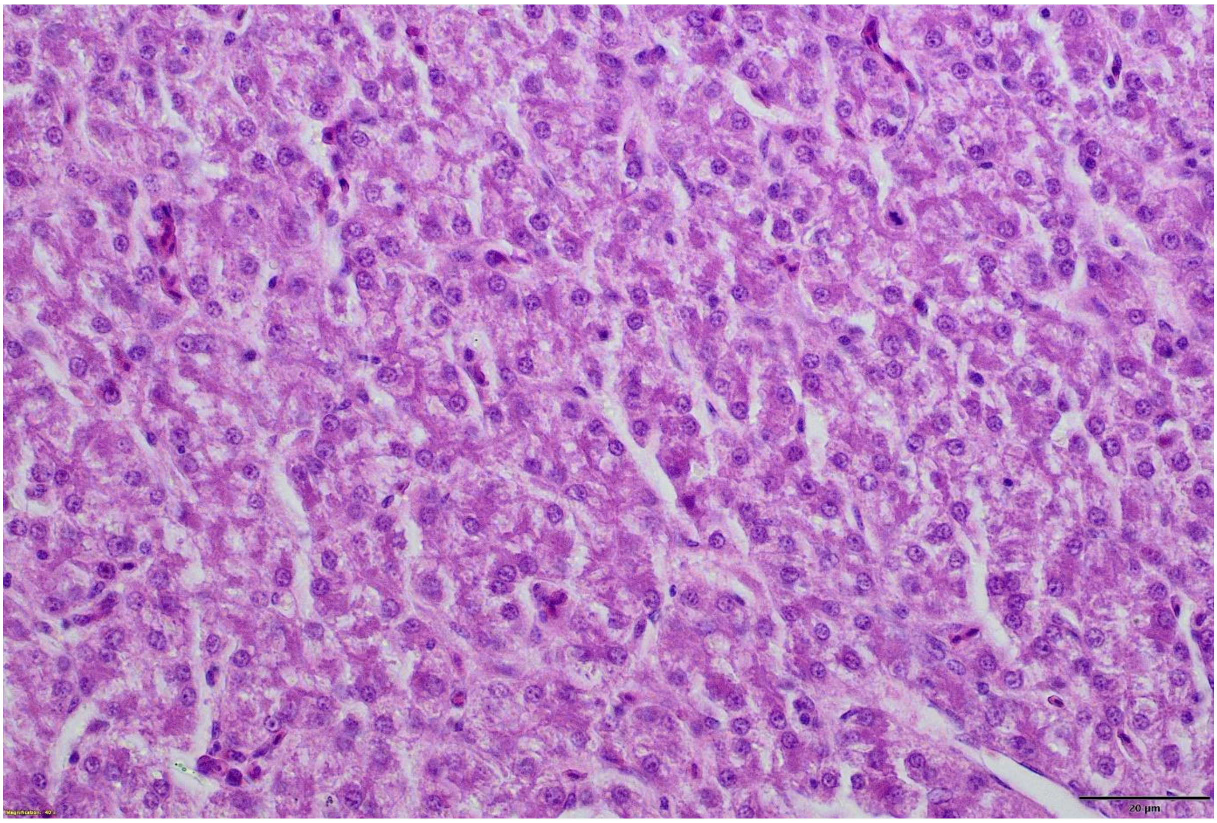

IoS_48 day 21

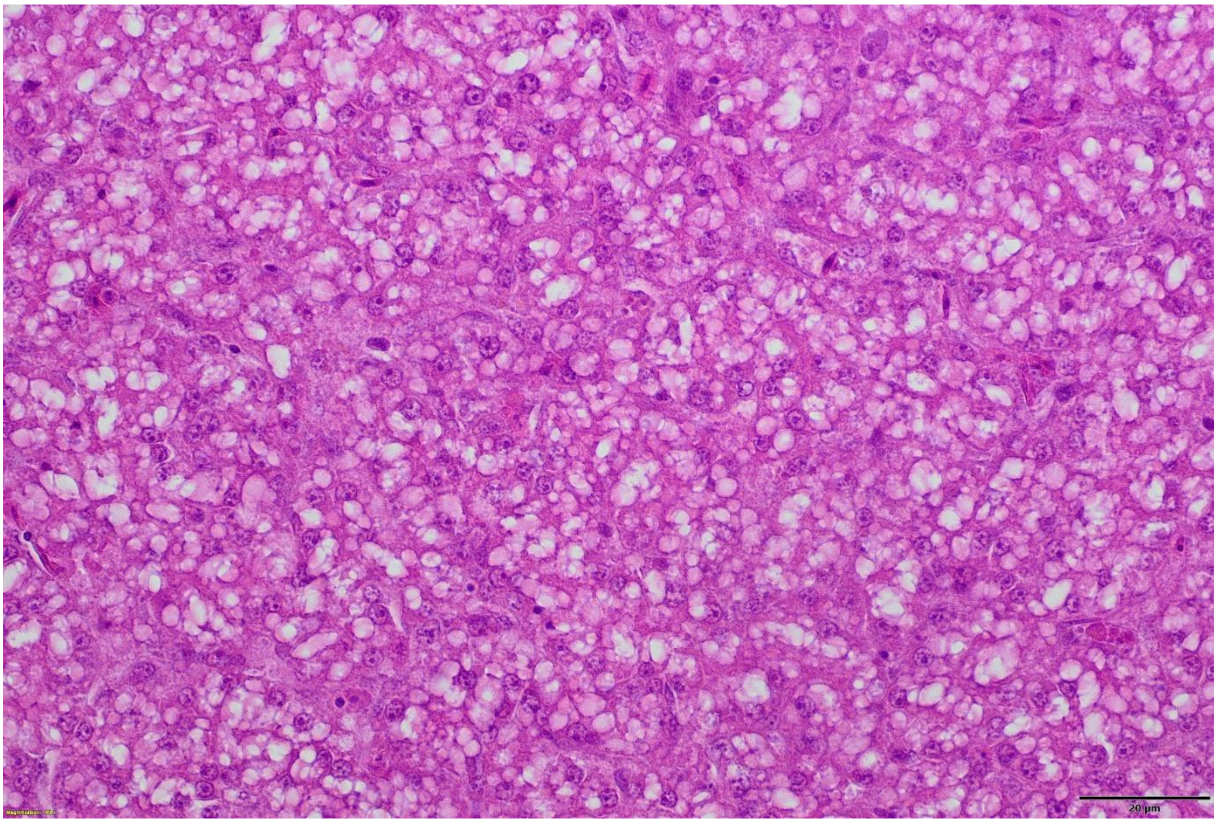

IoT_48 day 1

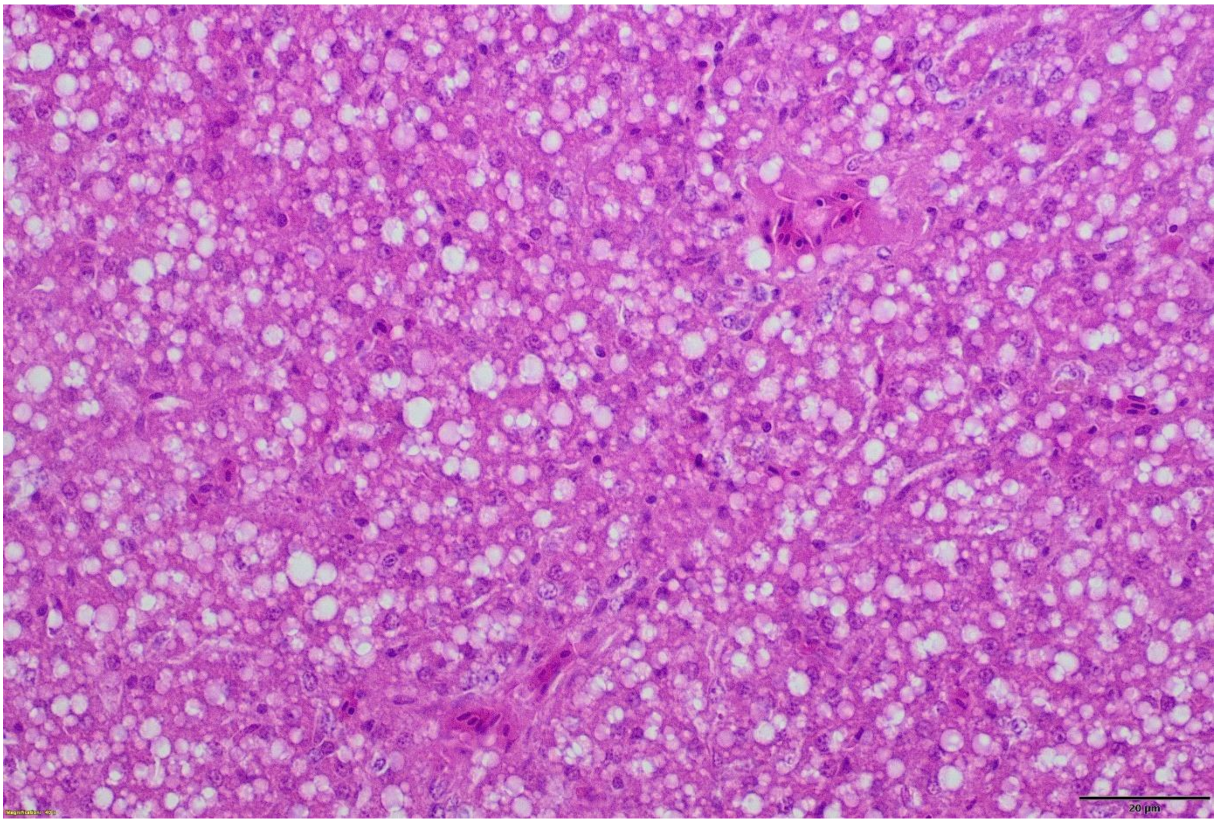

IoT_48 day 3

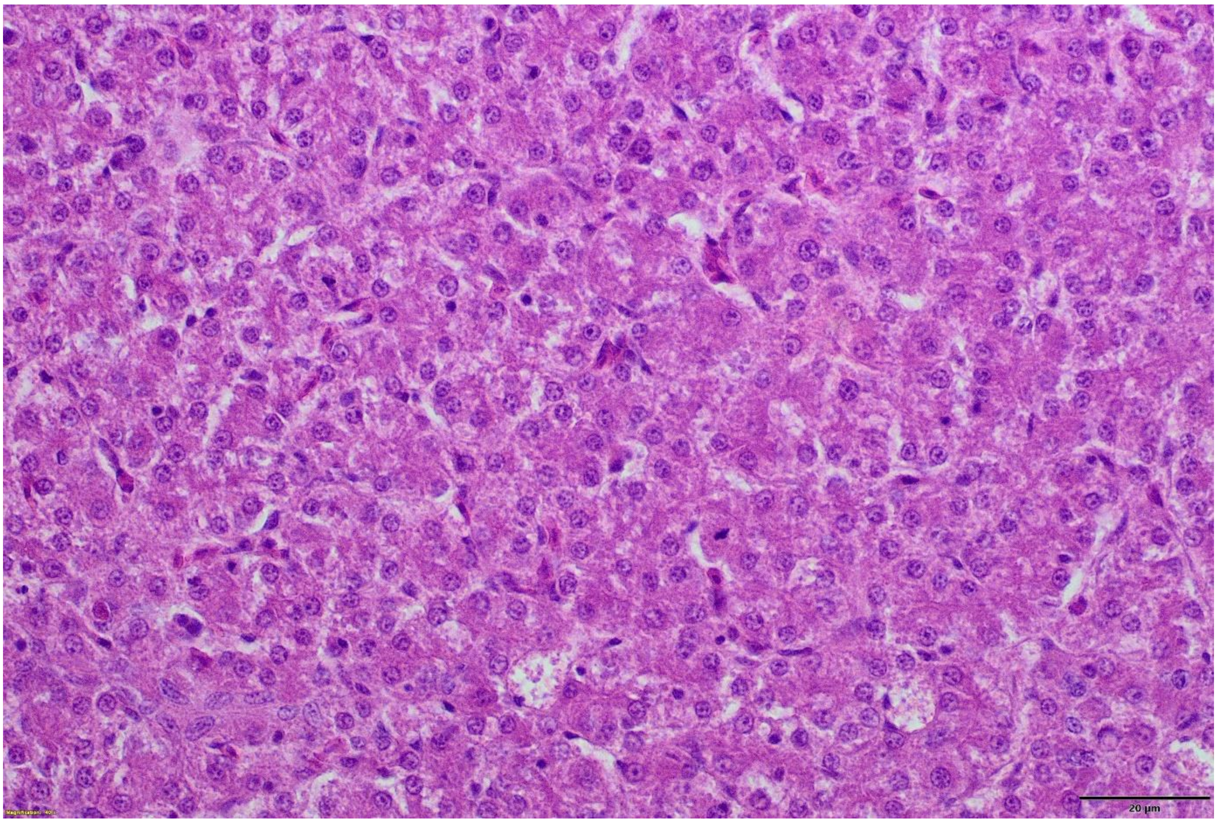

IoT_48 day 21

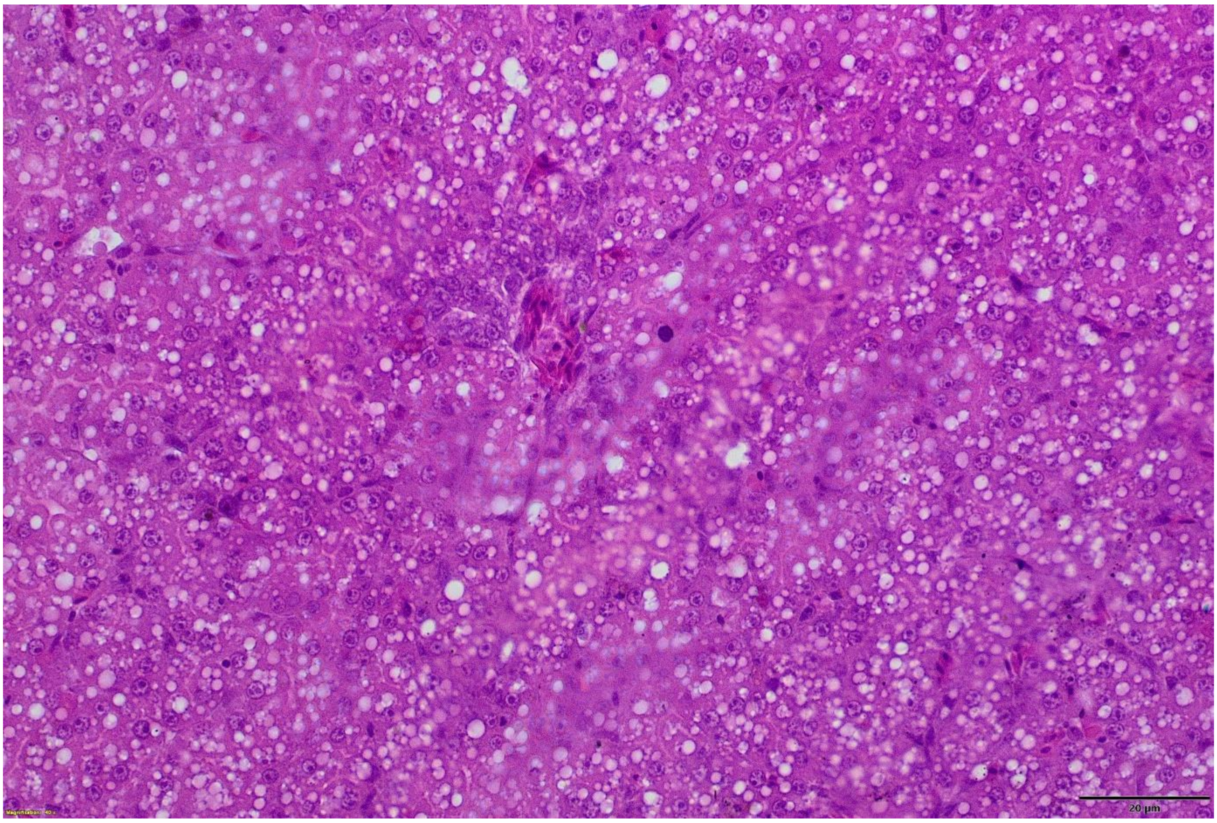

Int_G48 day 1

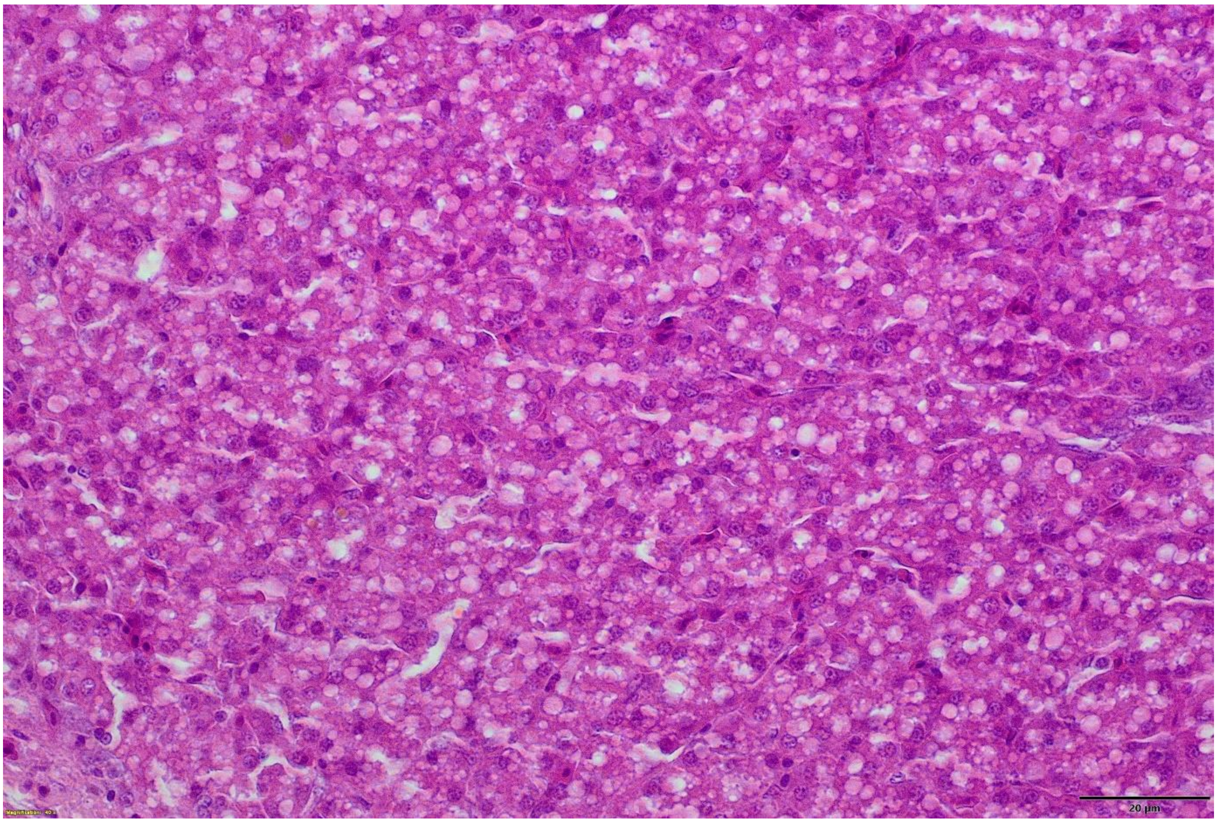

Int_G48 day 3

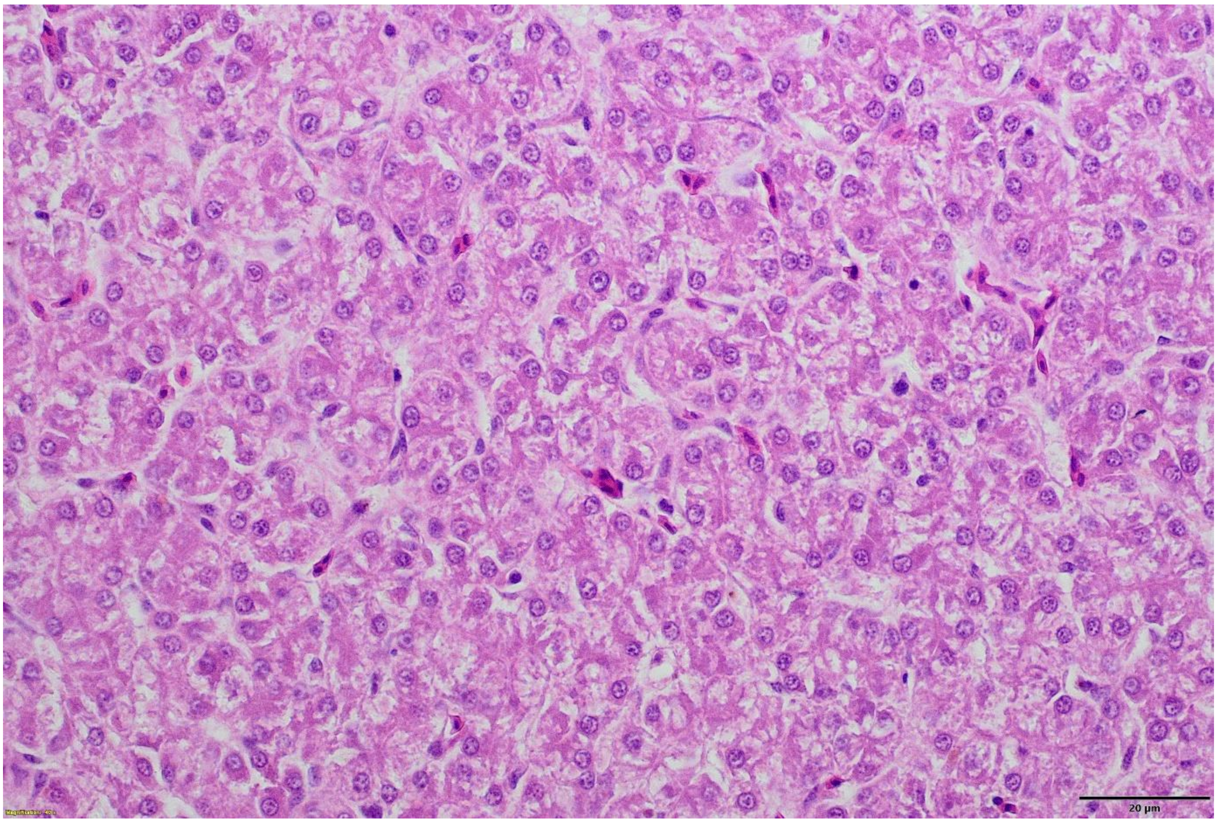

Int_G48 day 21

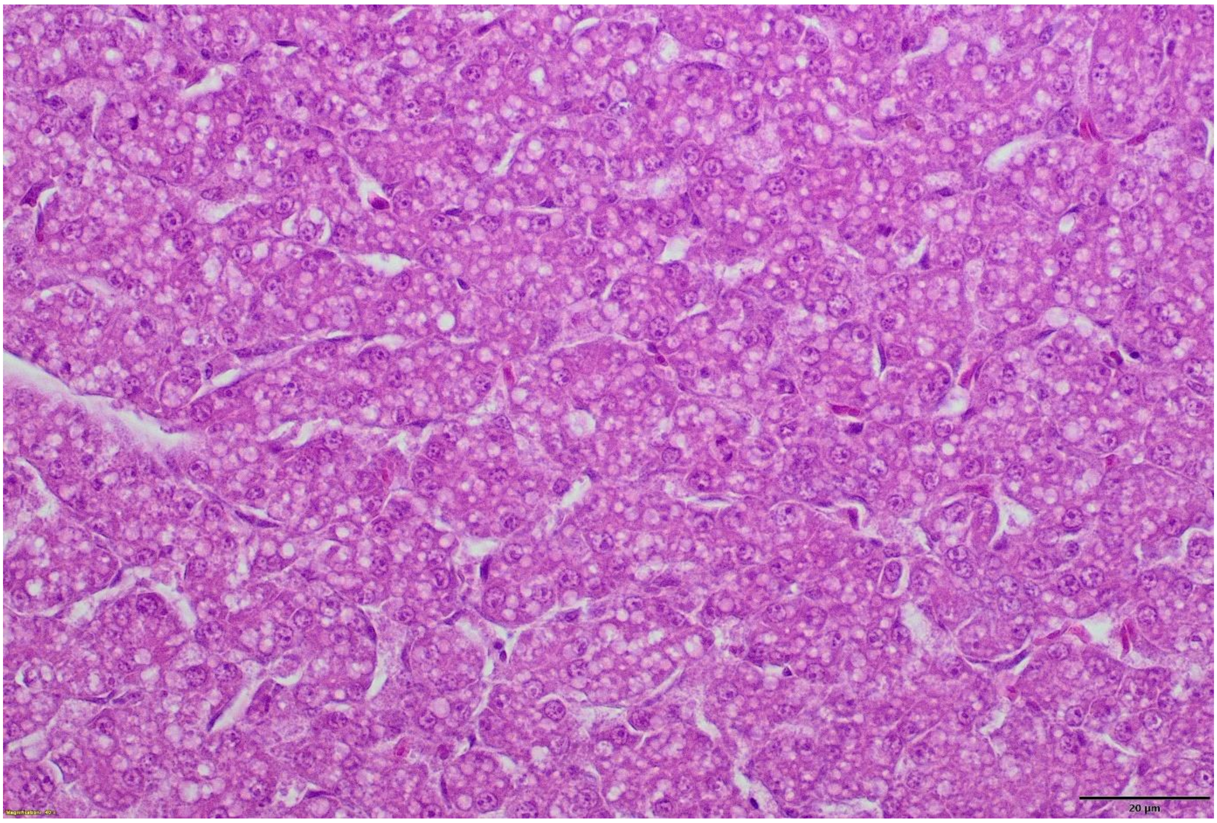

Int_GT48 day 1

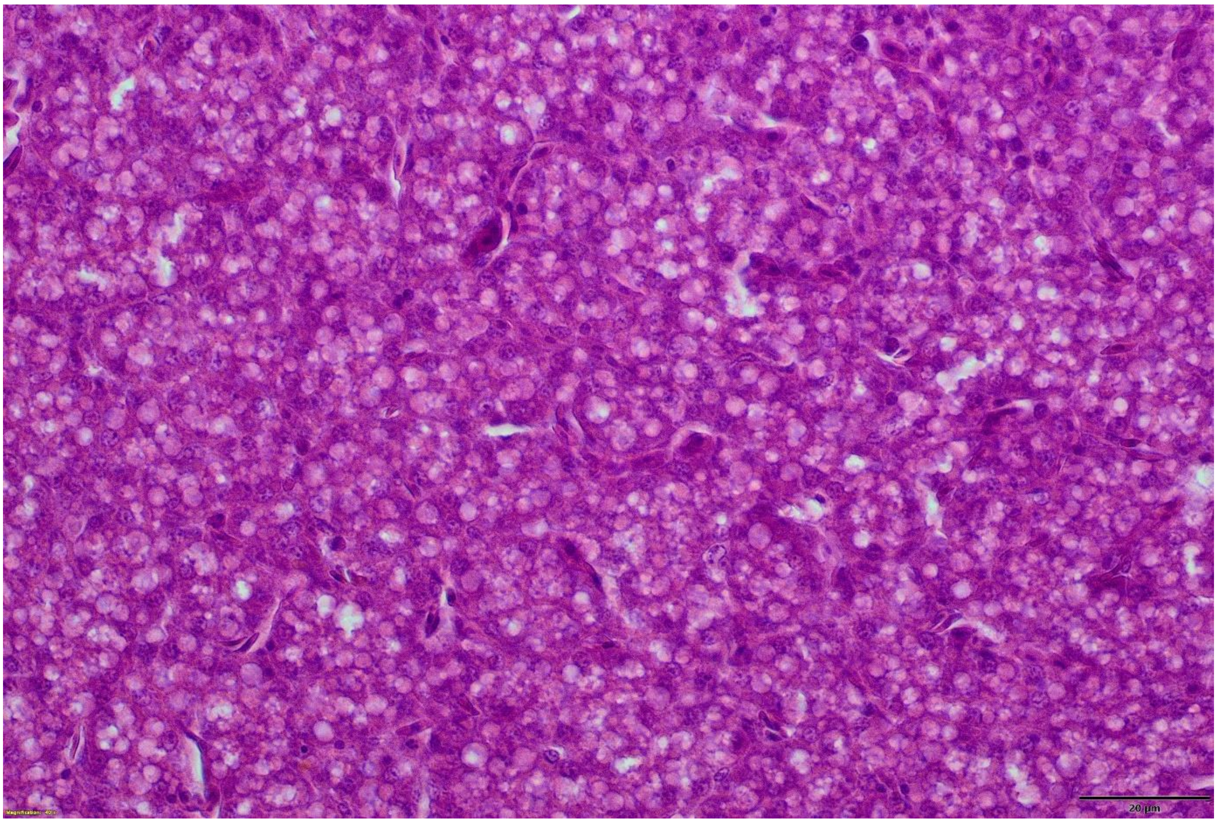

Int_GT48 day 3

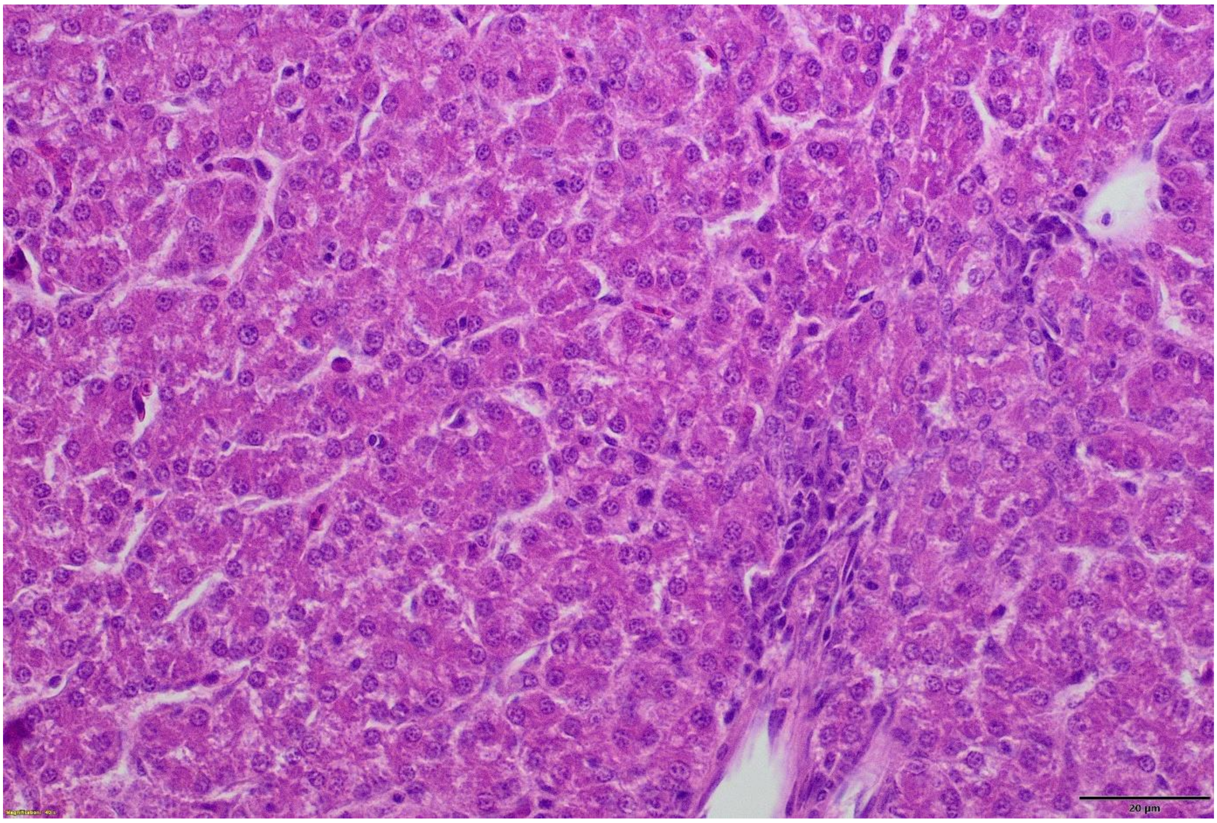

Int_GT48 day21
