# Supplementary material for: Impact of Threonine Supply in Early Ages on Gut Tissue Morphology, Liver Histology, and the Possible Changes in Leukocyte Numbers of Broilers
Source: Animals (Basel). 2025 Jan 27;15(3):370. doi: 10.3390/ani15030370 (PMC11815908; doi:10.3390/ani15030370)
Supplement: Supplementary file 1 [file animals-15-00370-s001.zip › Annex 2.pdf]

Annex 2

Representative images of alterations in the liver (2 images/alterations)

Mononuclear infiltration: Portal mononuclear cell infiltrations (lymphocytes, macrophages, and neutrophils), are accompanied by fibrosis which is a reaction to local depositioning of microorganisms and/or injury of local blood vessels from eg. toxic compounds (Kelly, 1993; MacLachlan and Cullen, 1995).

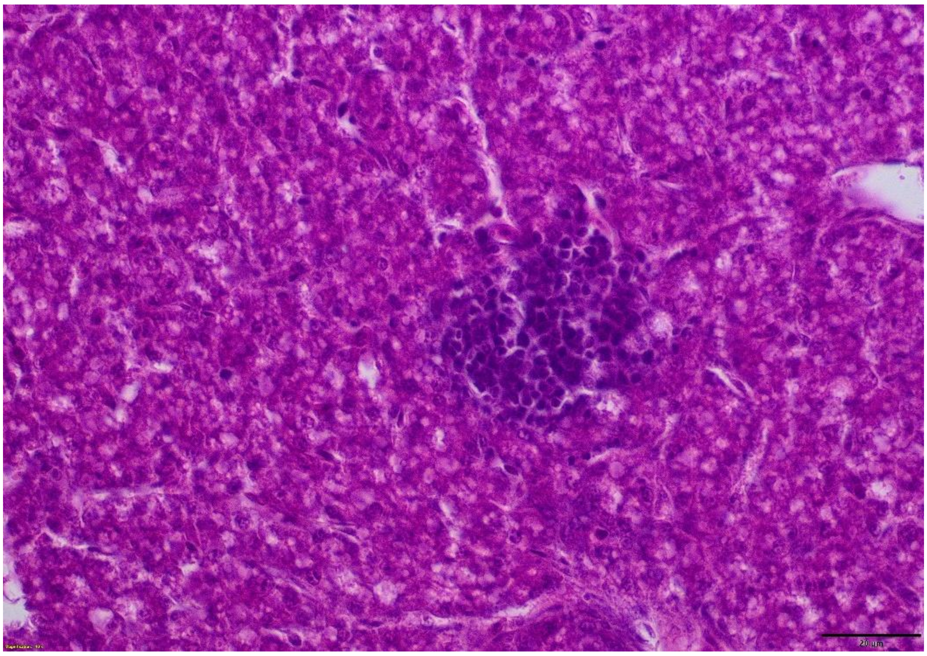

Int_0 day 3: Mononuclear cell infiltration

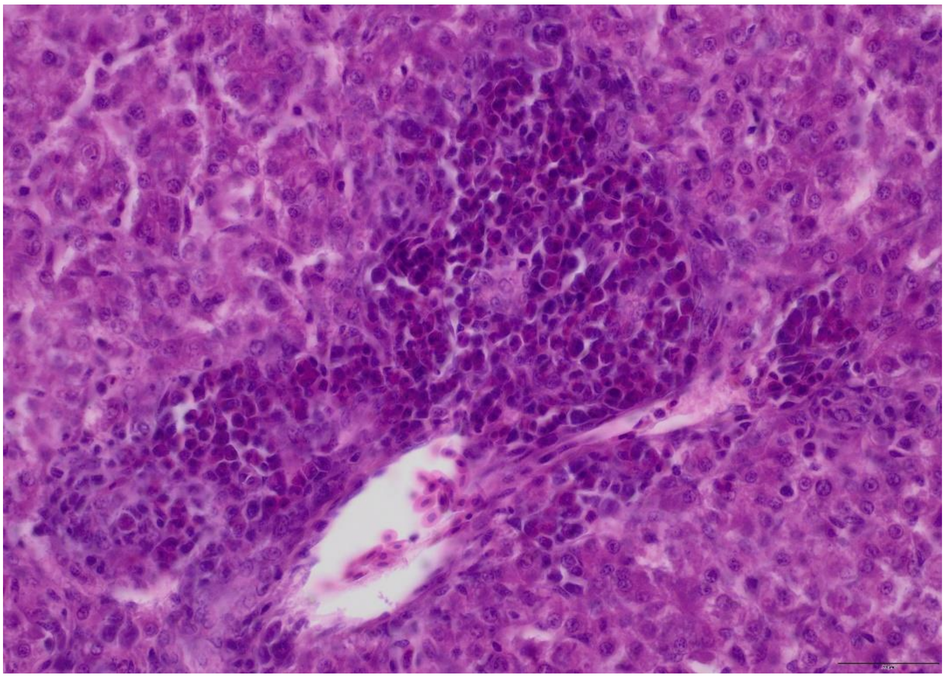

Int_48 day 3: Mononuclear cell infiltration

Heterophil granulocyte infiltration: Heterophils are the most numerous granulocytic leukocytes in the peripheral blood of poultry characterized by a multilobes nucleus and intracellular granules (Kogut, 2022)

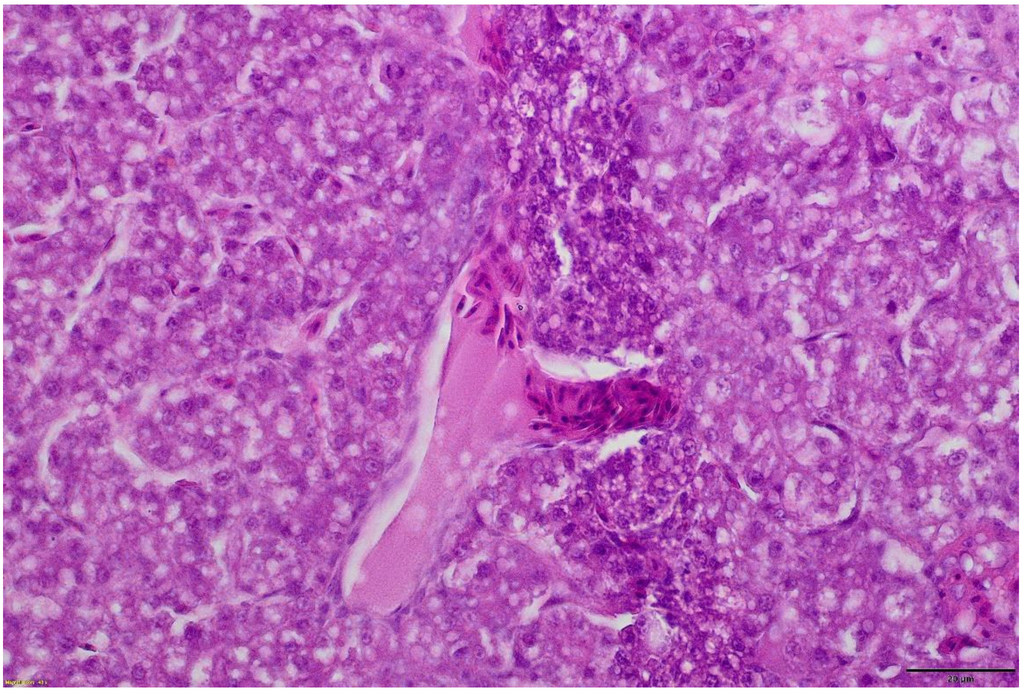

IoS_0 day 3: Heterophil granulocyte infiltration

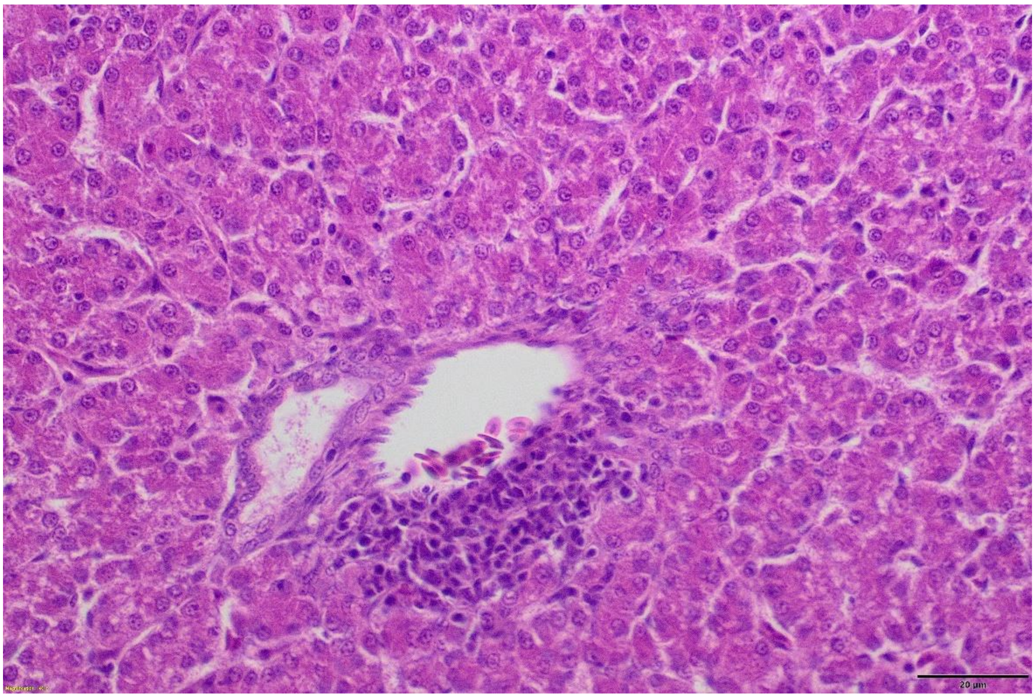

Int_GT48 day 21: Heterophil granulocyte infiltration

Lipid accumulation: (hepatocytic microvesicular lipid accumulation) was determined as foamy cytoplasm (Sonne, 2005) and more than 5% of fat in in the liver hepatocytes.

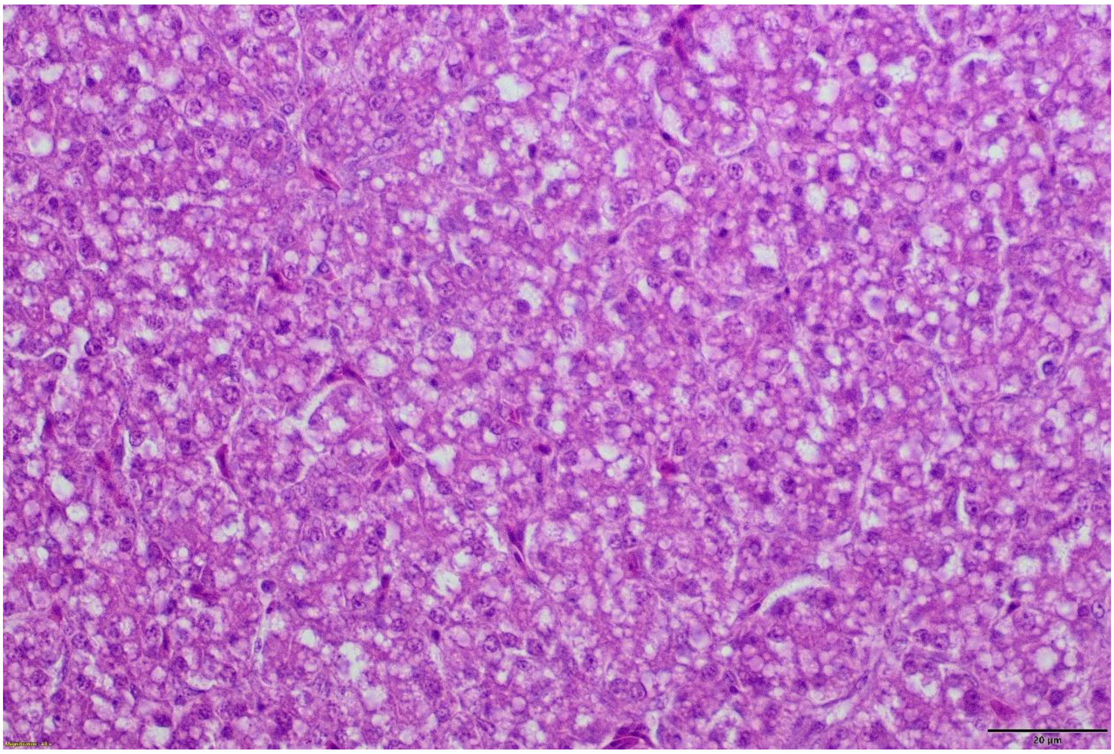

IoS_0 day 3: Lipid accumulation

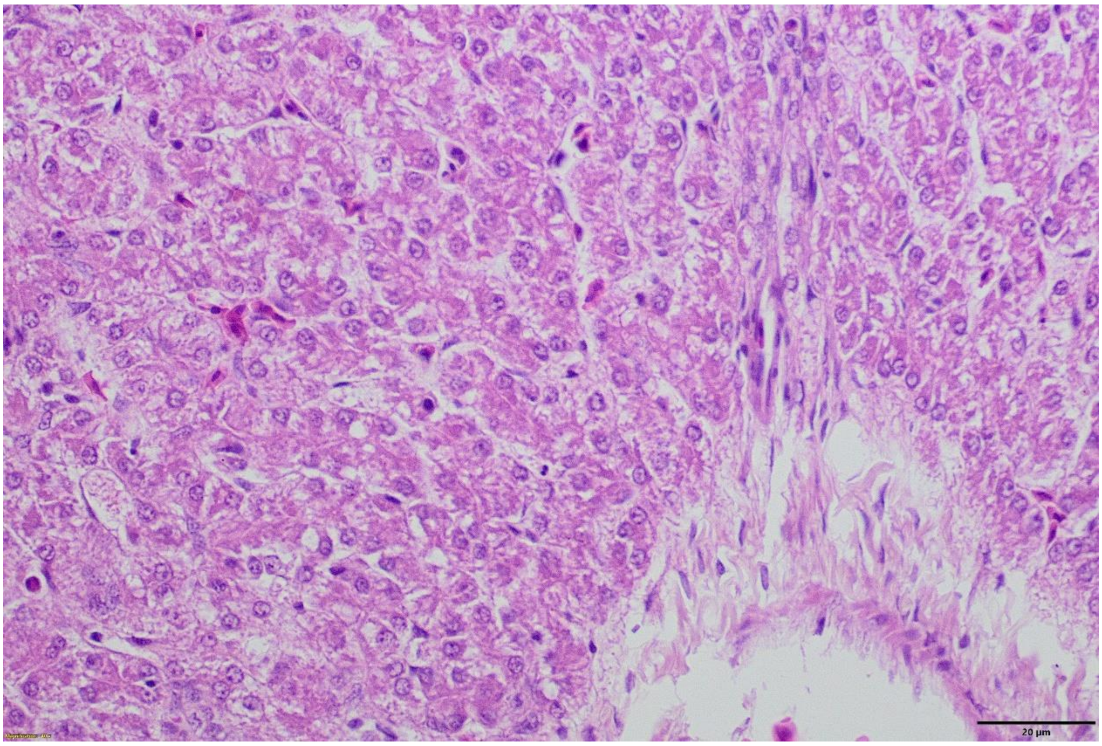

Int_G48 day 21_ Lipid accumulation

Vacuolisation: The vacuoles are generally rounded by a clear outline. In most cases they are filled with homogeneous eosinophilic material but occasionally there are empty areas within them (Skyes et al., 1975).

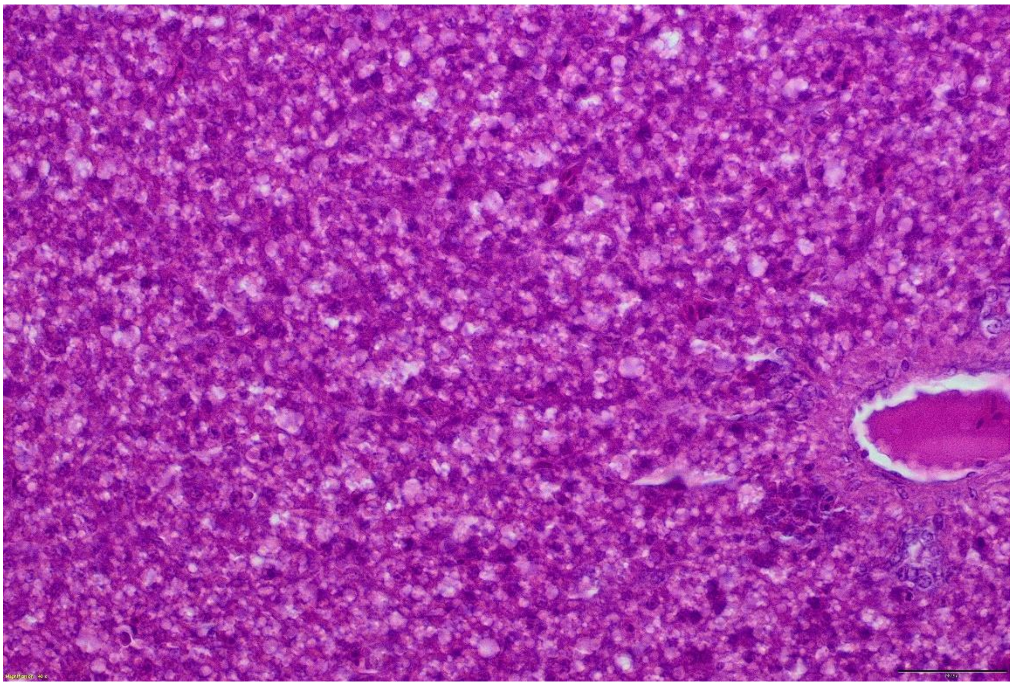

IoS_48 day 3: Vacuolization

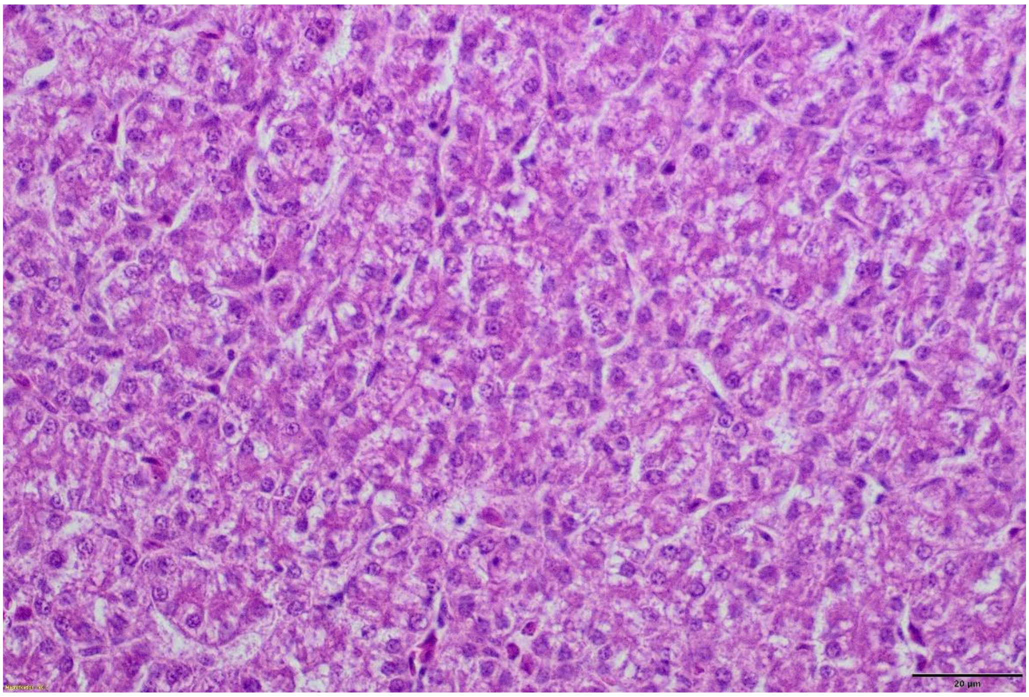

IoS_48 day 21: vacuolization

References

1. Sykes, B.I, Penny E, Purchase I.F.H, Hepatocyte vacuolation and increased liver weight occurring in anoxic rats, *Toxicology and Applied Pharmacology*,**1976,** 36:1,31-39, ISSN 0041-008X,
2. Sonne C., Dietz, R., Leifsson, P., Born, E., Letcher, R., Kirkegaard, M., & Muir, D., Riget, F., Hyldstrup, L. Do Organohalogen Contaminants Contribute to Liver Histopathology in East Greenland Polar Bears. *Env Health Persp*. **2005,** 113. 1569-74. 10.1289/ehp.8038.
3. MacLachlan N.J, Cullen J.M. Liver, biliary system and exocrine pancreas. *Thomsons Special Veterinary Pathology*. **1995,** (Carlton WW, Donald McGavin M, eds). St. Louis, MO:Mosby Year Book, 81–115.
4. Kelly W.R. The liver and biliary system. *Pathology of Domestic Animals* **1993,** 319–406
5. Kogut M.H. Avian Immunology (Third Edition),Subchapter 8.2 - Avian granulocytes, *Academic Press,* **2022,** Editor(s): Bernd Kaspers, Karel A. Schat, Thomas W. Göbel, Lonneke Vervelde, 197-203,
