# Supplementary material for: Impact of Threonine Supply in Early Ages on Gut Tissue Morphology, Liver Histology, and the Possible Changes in Leukocyte Numbers of Broilers
Source: Animals (Basel). 2025 Jan 27;15(3):370. doi: 10.3390/ani15030370 (PMC11815908; doi:10.3390/ani15030370)
Supplement: Supplementary file 1 [file animals-15-00370-s001.zip › Annex 3.pdf]

# Annex 3

## Representative images of transversal sections of gut histomorphology in each treatment group from the duodenum, ileum and colon on days 1, 3 and 21.

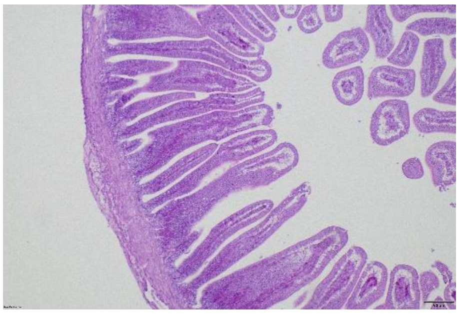
Int_0 day 1 duodenum

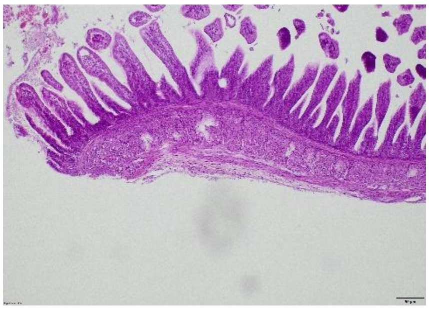
Int_0 day 1 ileum

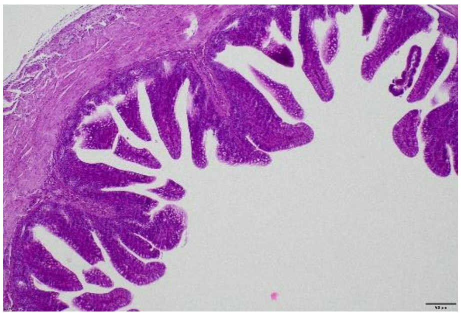
Int_0 day 1 colon

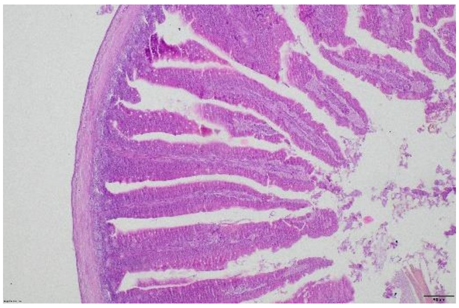
Int_0 day 3 duodenum

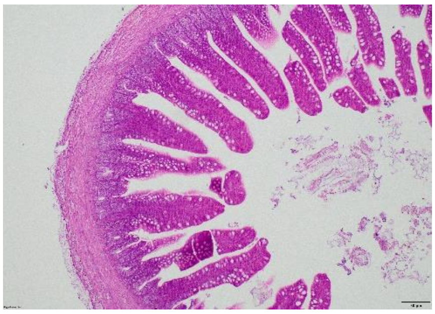
Int_0 day 3 ileum

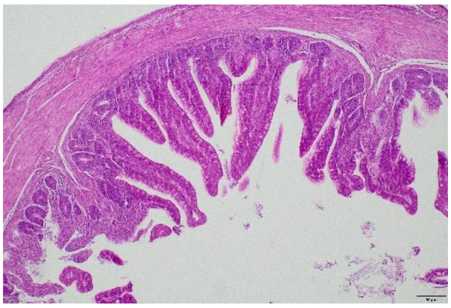
Int_0 day 3 colon

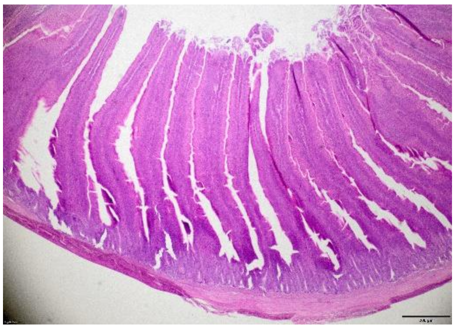
Int_0 day 21 duodenum

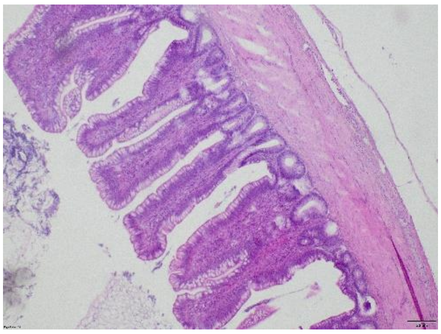
Int_0 day 21 ileum

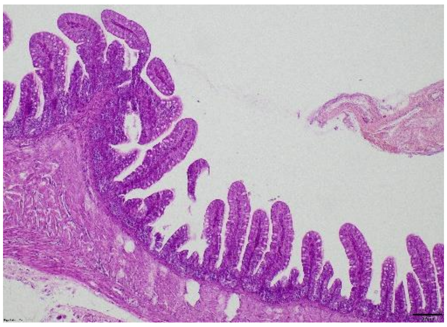
Int_0 day 21 colon

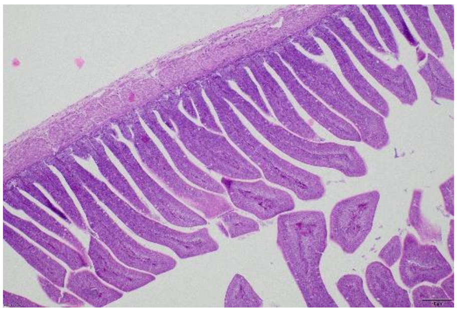
IoS_0 day 1 duodenum

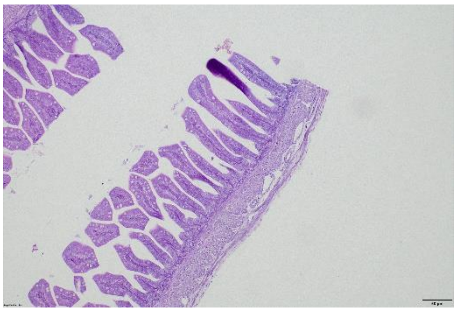
IoS_0 day 1 ileum

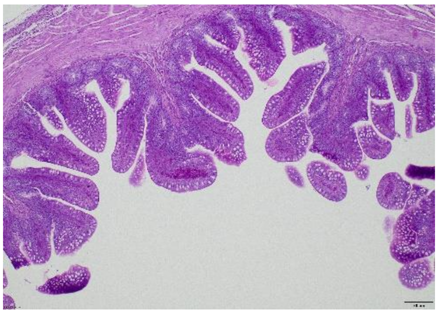
IoS_0 day 1 colon

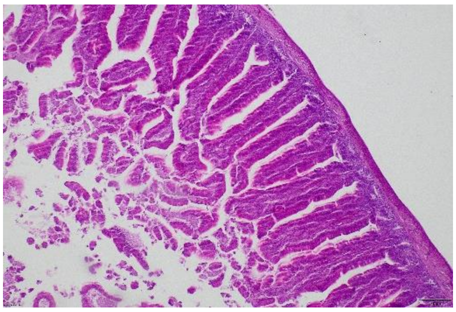

IoS_0 day 3 duodenum

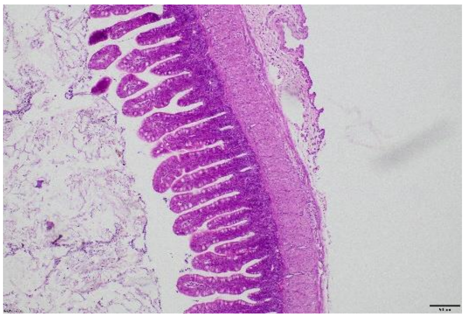

IoS_0 day 3 ileum

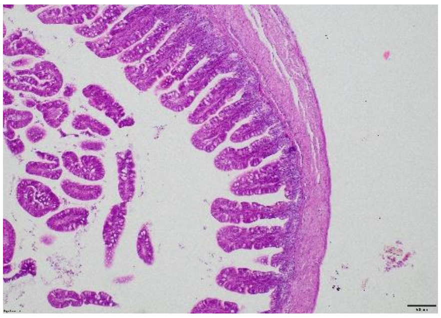

IoS_0 day 3 colon

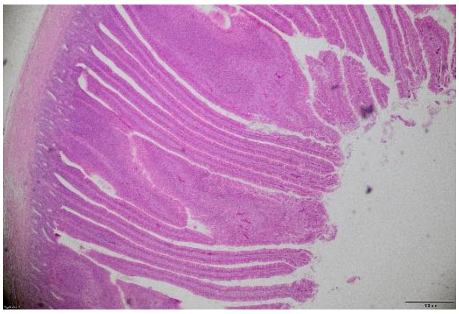

IoS_0 day 21 duodenum

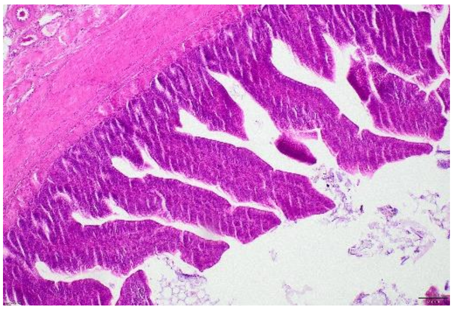

IoS_0 day 21 ileum

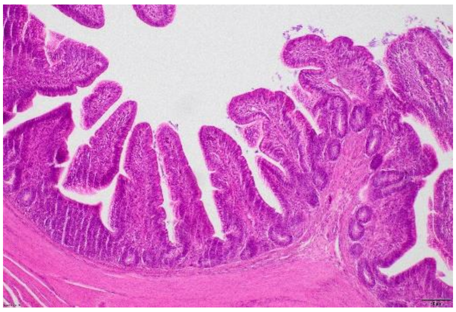

IoS_0 day 21 colon

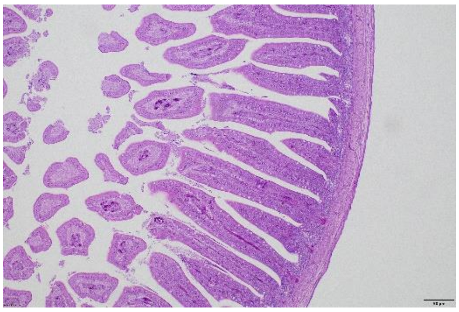

Int_48 day 1 duodenum

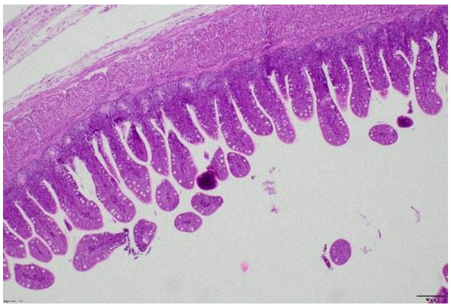

Int_48 day 1 ileum

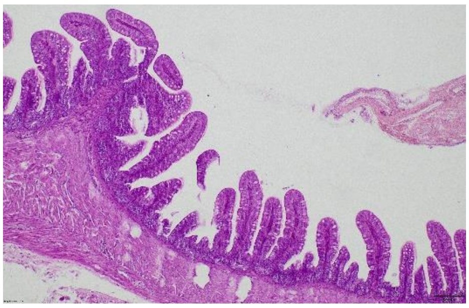

Int_48 day 1 colon

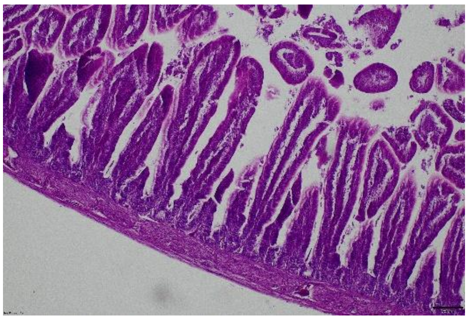

Int_48 day 3 duodenum

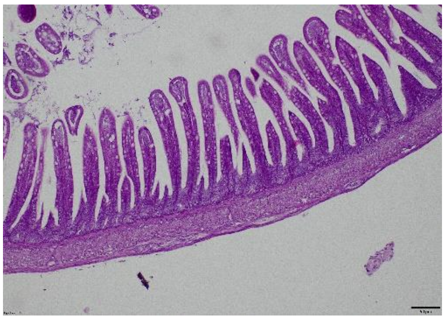

Int_48 day 3 ileum

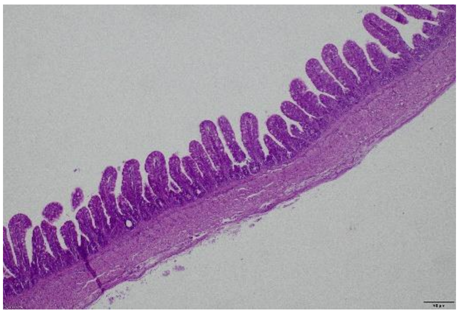

Int_48 day 3 colon

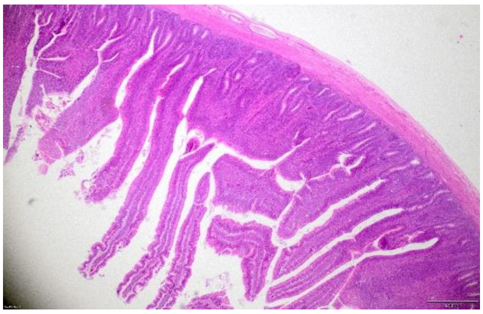

Int_48 day 21 duodenum

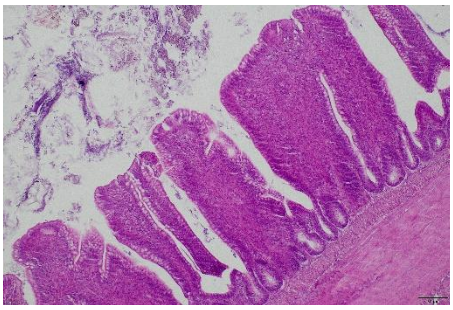

Int_48 day 21 ileum

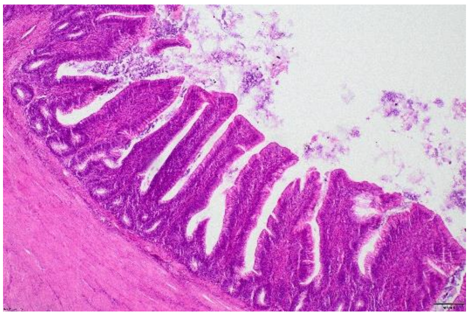

Int_48 day 21 colon

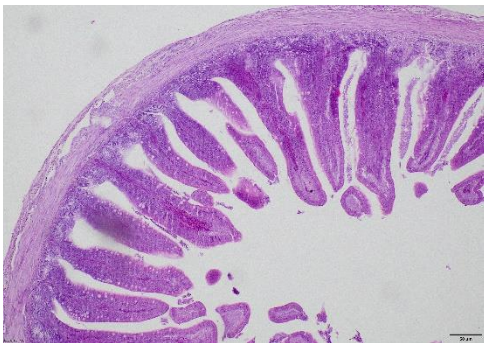

IoS_48 day 1 duodenum

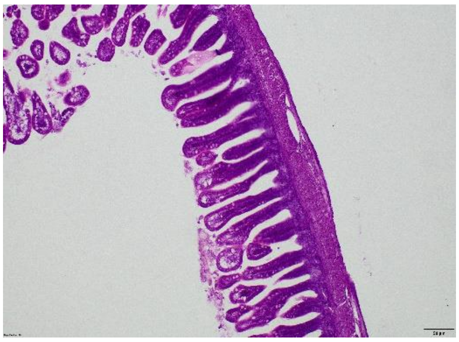

IoS_48 day 1 ileum

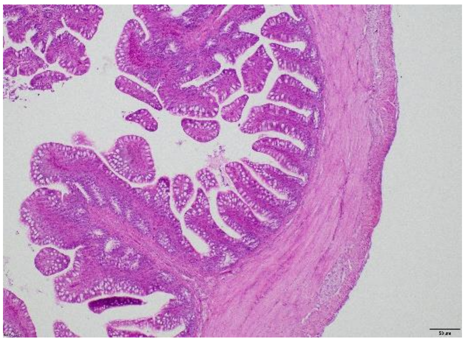

IoS_48 day 1 colon

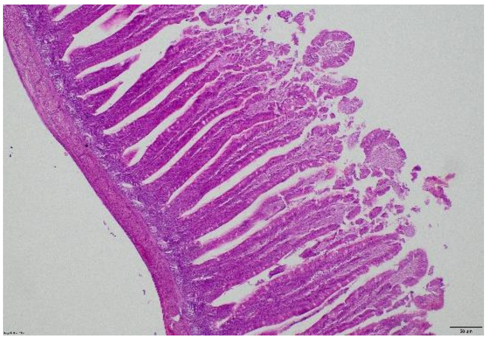

IoS_48 day 3 duodenum

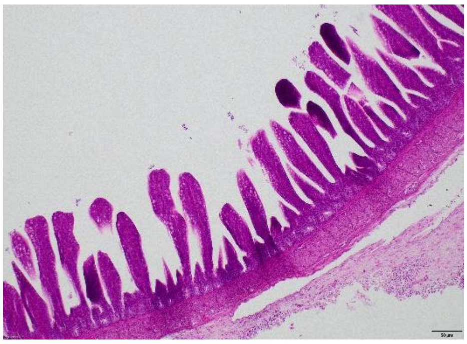

IoS_48 day 3 ileum

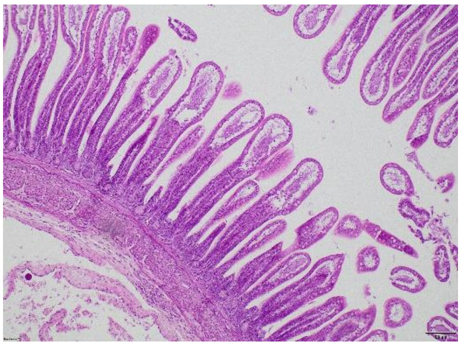

IoS_48 day 3 colon

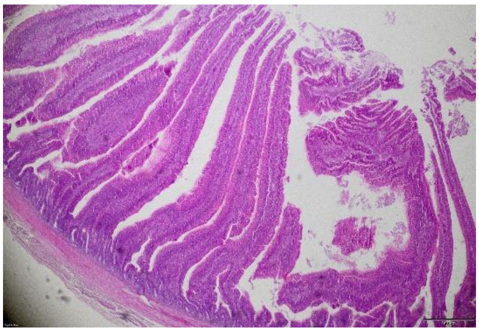

IoS_48 day 21 duodenum

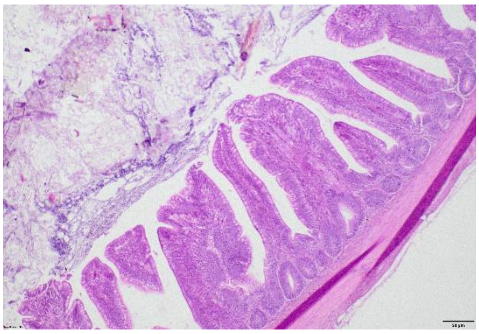

IoS_48 day 21 ileum

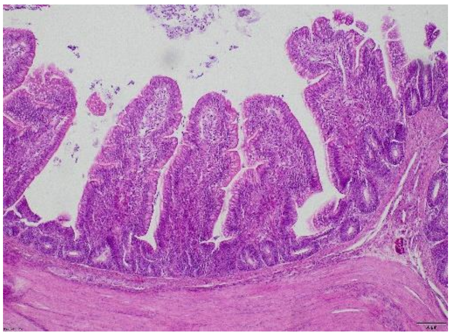

IoS_48 day 21 colon

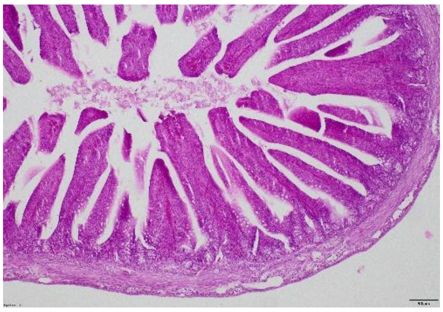
IoT_48 day 1 duodenum

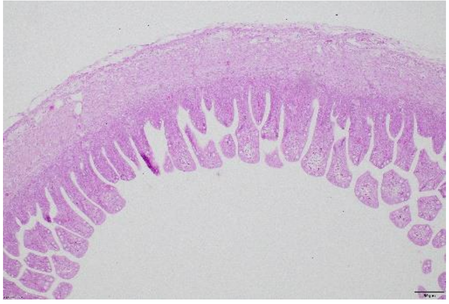
IoT_48 day 1 ileum

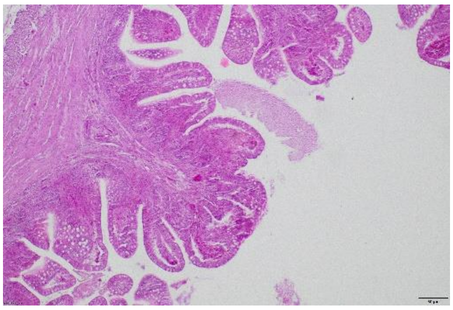
IoT_48 day 1 colon

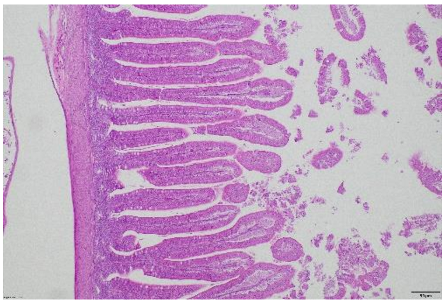
IoT_48 day 3 duodenum

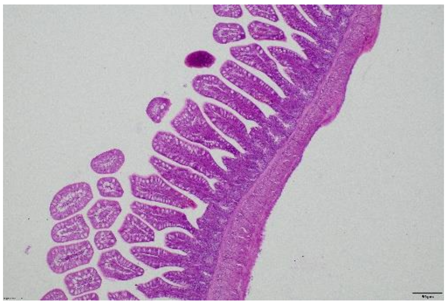
IoT_48 day 3 ileum

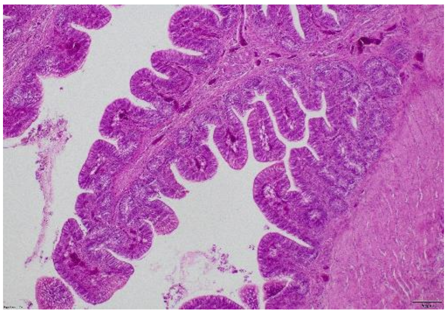
IoT_48 day 3 colon

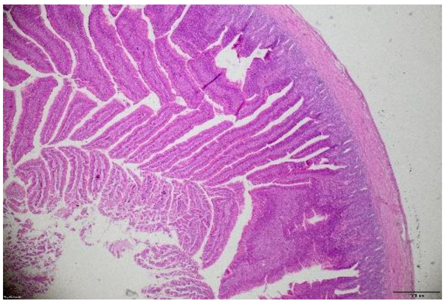
IoT_48 day 21 duodenum

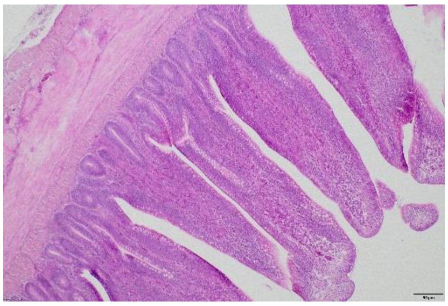
IoT_48 day 21 ileum

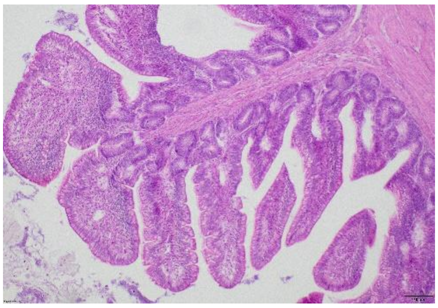
IoT_48 day 21 colon

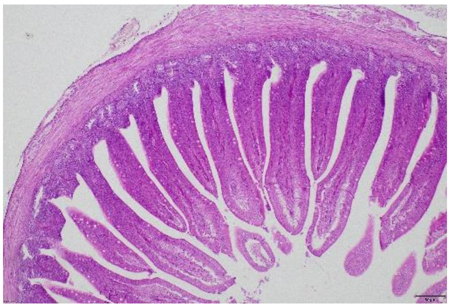
Int_G48 day 1 duodenum

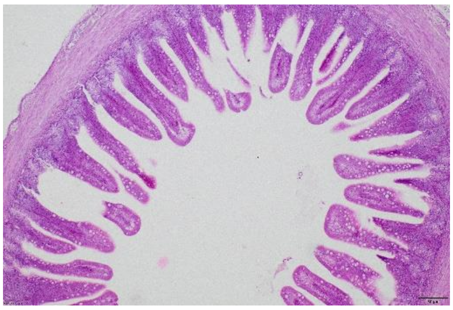
Int_G48 day 1 ileum

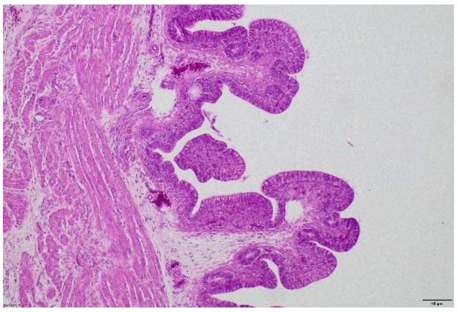
Int_G48 day 1 colon

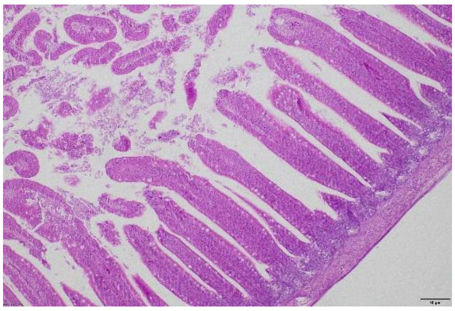
Int_G48 day 3 duodenum

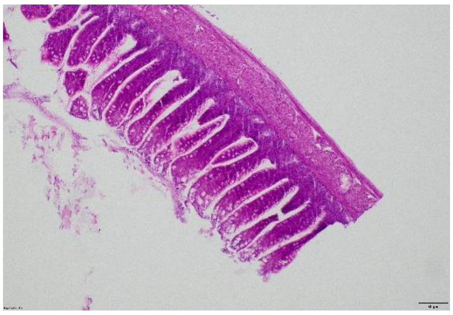
Int_G48 day 3 ileum

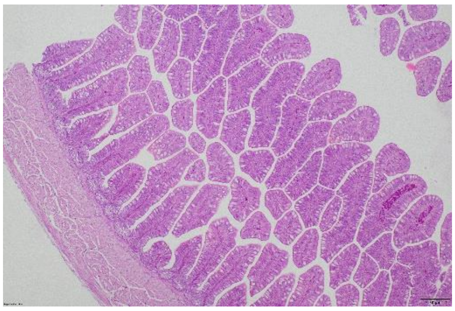
Int_G48 day 3 colon

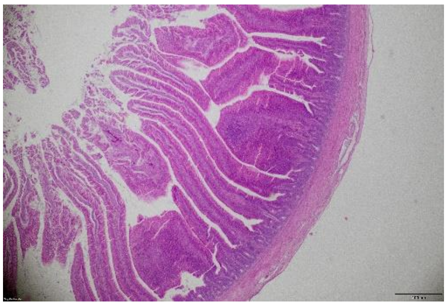
Int_G48 day 21 duodenum

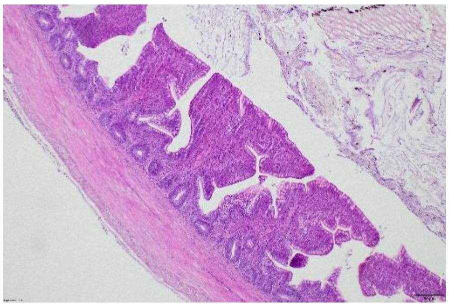
Int_G48 day 21 ileum

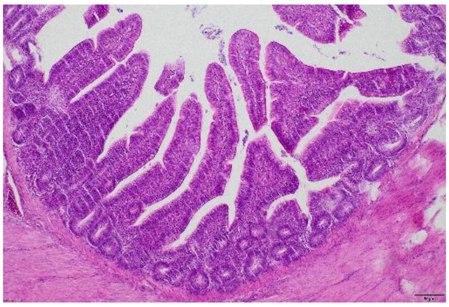
Int_G48 day 21 colon

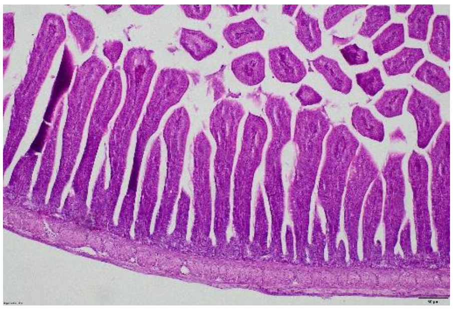
Int_GT48 day 1 duodenum

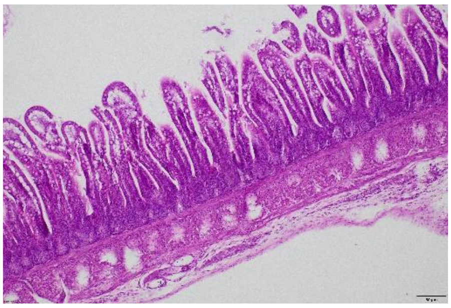
Int_GT48 day 1 ileum

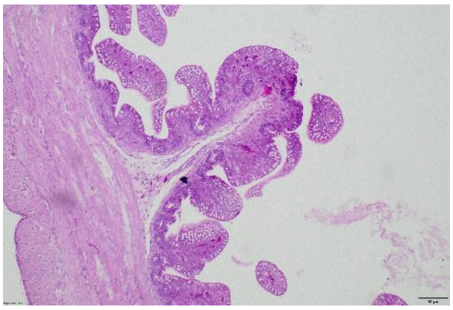
Int_GT48 day 1colon

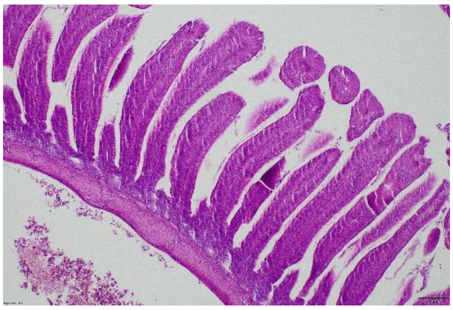
Int_GT48 day 3 duodenum

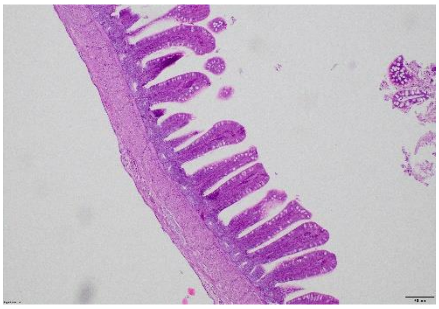
Int_GT48 day 3 ileum

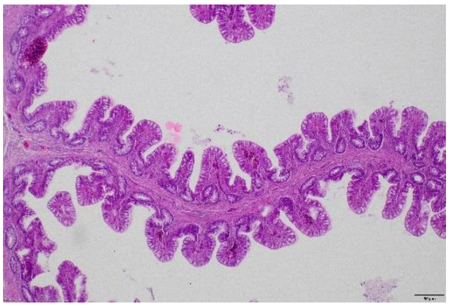
Int_GT48 day 3 colon

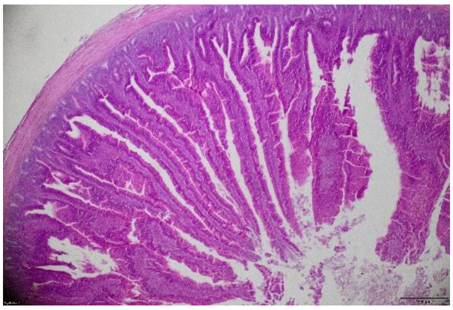
Int_GT48 day 21 duodenum

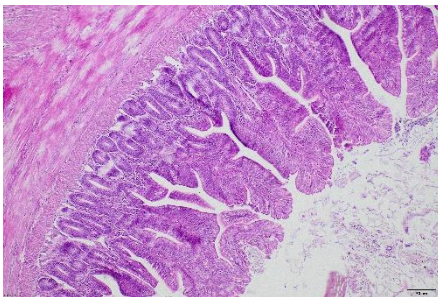
Int_GT48 day 21 ileum

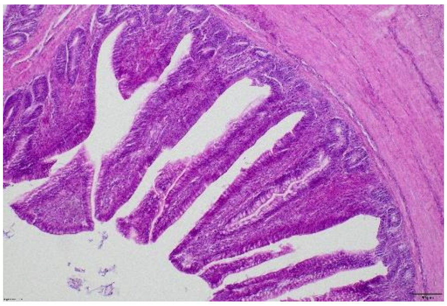
Int_GT48 day 21 colon
